# Antimicrobial Resistance in Farm Animals in Brazil: An Update Overview

**DOI:** 10.3390/ani10040552

**Published:** 2020-03-26

**Authors:** Renata F. Rabello, Raquel R. Bonelli, Bruno A. Penna, Julia P. Albuquerque, Rossiane M. Souza, Aloysio M. F. Cerqueira

**Affiliations:** 1 Departamento de Microbiologia e Parasitologia, Universidade Federal Fluminense, Niterói 24210-130, Brazilbpenna@id.uff.br (B.A.P.); jp_albuquerque@id.uff.br (J.P.A.); 2 Instituto de Microbiologia Paulo de Góes, Universidade Federal do Rio de Janeiro, Rio de Janeiro 21941-902, Brazil; 3 Centro Estadual de Pesquisa em Sanidade Animal, Empresa de Pesquisa Agropecuária do Estado do Rio de Janeiro, Niterói 24120-191, Brazil

**Keywords:** antimicrobial resistance, poultry, pork, beef cattle, dairy cattle, Brazil

## Abstract

**Simple Summary:**

Beyond their use of treating human and animal diseases, antimicrobial agents have also been employed in animal feeding as “growth promoters”, being administrated at low doses throughout the husbandry period, and leading to beneficial effects, mainly for large-scale production. However, the consequent selective pressure has helped to increase antimicrobial resistance frequencies in isolates from animals globally. Brazil is a major food producer and exporter; therefore, it is of great value to look at the panorama of bacterial resistance associated with farm animals. Since 1998, many drugs have been prohibited from being used as growth promoters in Brazil. However, neither data on the prophylactic and therapeutic use of antimicrobial agents in farms, nor results of contemporary and integrated surveillance programs regarding antimicrobial resistance associated with food production animals, are still publicly available. This review aims to update and discuss the available Brazilian data on this topic emphasizing legal aspects, occurrence, and genetics of the resistance reported by studies published since 2009, focusing on producing animals and derived foods with the most global public health impact. Data here compiled may be useful to monitor and evaluate the local situation and serve as a basis for establishing parameters for the future.

**Abstract:**

In animal husbandry, antimicrobial agents have been administered as supplements to increase production over the last 60 years. Large-scale animal production has increased the importance of antibiotic management because it may favor the evolution of antimicrobial resistance and select resistant strains. Brazil is a significant producer and exporter of animal-derived food. Although Brazil is still preparing a national surveillance plan, several changes in legislation and timely programs have been implemented. Thus, Brazilian data on antimicrobial resistance in bacteria associated with animals come from official programs and the scientific community. This review aims to update and discuss the available Brazilian data on this topic, emphasizing legal aspects, incidence, and genetics of the resistance reported by studies published since 2009, focusing on farm animals and derived foods with the most global public health impact. Studies are related to poultry, cattle, and pigs, and mainly concentrate on non-typhoid *Salmonella*, *Escherichia coli*, and *Staphylococcus aureus*. We also describe legal aspects of antimicrobial use in this context; and the current occurrence of genetic elements associated with resistance to beta-lactams, colistin, and fluoroquinolones, among other antimicrobial agents. Data here presented may be useful to provide a better understanding of the Brazilian status on antimicrobial resistance related to farm animals and animal-derived food products.

## 1. Introduction

**Figure A1:**
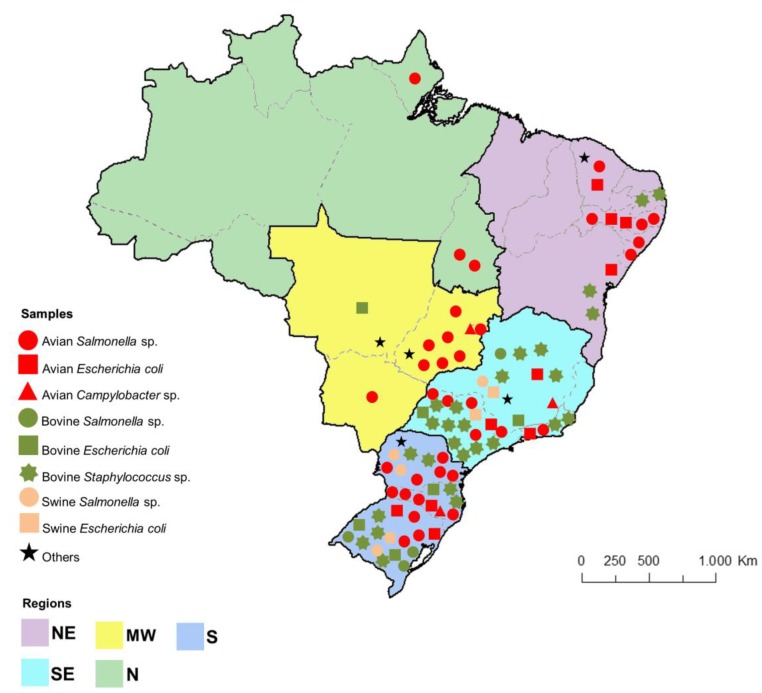
Geographic regions of Brazil and distribution of data, published from 2010 to 2019.

The increase in the world population will lead to a significant increase in food production demand in the coming years (i.e., in the order of 60% to nearly 10 billion people by the year 2050) [1]. As a large and tropical country, Brazil represents one of the biggest global food producers. The country is particularly relevant when it comes to increasing food supply, as it is a leading country in animal production, both for domestic consumption and for export to many countries. Furthermore, as a continental country divided into five distinct regions (N, North; NE, Northeast; MW, Midwest; SE, Southeast, and S, South; Figure A1), Brazil has particular aspects related to animal production. In general, most of the beef production for foreign markets comes from Midwestern states, while pork and poultry production is concentrated in Southern states. Otherwise, dairy and egg production, which is mainly destined for the domestic market, is usually concentrated near large urban centers throughout the country.

The increase in animal production and productivity has a vital link with the control of infectious and parasitic diseases, especially in tropical countries. Thus, therapeutic and rational use of antimicrobial agents is inevitable, especially in intensive farming animals, such as swine, poultry, and confined beef cattle. On the other hand, concerning the use of antimicrobial agents, as growth promoters, their contribution (to a lower occurrence of diseases and higher productivity) has gradually been abolished by their evident participation in the selection of resistant microorganisms that can reach humans through the food chain [2,3,4,5].

The increase in antimicrobial resistance is a growing issue worldwide, and is associated with several factors; therefore, it requires broad and coordinated action to contain or diminish the problem. In a One Health perspective, there is a consensus that various actors must work together to control the increased antimicrobial resistance worldwide. Regarding animal production, this implies changes in some traditional farming practices [6,7].

Due to its relevance in animal food production, the way Brazil regulates the use of antimicrobial agents in animals, whether for prophylactic or therapeutic use, may have implications at the local and global levels of antimicrobial resistance. In addition to legal issues, the Brazilian scientific community has been searching for the profile and evolution of antimicrobial resistance of bacterial pathogens isolated from several animals and foods. Furthermore, some international publications have sought and included data related to Brazil. Although numerous, these studies are often focused on a small collection of samples or isolates obtained in a restricted geographic region; thus, jeopardizing their representativity. 

The present overview intends to update and discuss the available Brazilian data on this topic, emphasizing legal aspects, evolution, and genetics of the antimicrobial resistance focused on producing animals and derived foods with global public health impact. Moreover, due to their relevance as bacterial pathogens, non-typhoid *Salmonella* sp., *Escherichia coli, Campylobacter* spp., and *Staphylococcus aureus* are emphasized. Some data on other animals, as well as other bacterial pathogens, are briefly presented. The data presented here mainly covers the last decade; we considered only studies that have references to the date, location of collection, and methodology employed. When a specific state was mentioned as a place of sampling, its region was referred (abbreviated in brackets). Since the recommendations and interpretation standards for the reading of susceptibility tests have varied during this period, and their standardization was not applicable, data that met previous criteria were treated equally and compared.

## 2. Legal Aspects Related to Animal Antimicrobial Control and Monitoring Programs in Brazil

The burden of antimicrobial resistance has led to greater control in the use of antimicrobial agents in animal production (as growth promoters and for therapeutic purposes). In this sense, the European Union has progressively restricted the use of antimicrobial agents as additives to improve zootechnical performance in producing animals, including the prohibition of the use of these drugs since 2006 [8]. Particularly in intensive farming animals, the mandatory sale of medically important antimicrobials for therapeutic use, only in animals with a veterinary prescription, was instituted between 2017 and 2018 [9,10]. 

To meet international requirements, Brazil has gradually established, through various legal regulations, a greater rigor regarding antibiotic use and other performance-enhancing additives (Table A1). Thus, the use of avoparcin was prohibited in 1998; antimonial compounds in 2002; chloramphenicol and nitrofurans (including veterinary clinical use) in 2003; olaquindox in 2004; carbadox in 2005, amphenicols, tetracyclines, beta-lactams (benzylpenicillin and cephalosporins), quinolones, and sulfonamides in 2009; spiramycin and erythromycin in 2012; and colistin in 2016 [11,12,13,14,15,16,17,18,19]. Recently, the use of the additives tylosin, lincomycin, and tiamulin was prohibited. Virginiamycin and bacitracin are the remaining additives allowed for use [20].

The World Health Organization (WHO) elected the essential antimicrobial agents for human medicine, as a reference to assist the formulation and prioritization of risk analysis and management strategies in order to contain antimicrobial resistance. Antimicrobial agents important for human medicine are classified by WHO according to established criteria as critically important, highly important, and important. However, even antimicrobial agents used only in animals (apramycin, ceftiofur, tylosin, enrofloxacin, and florfenicol) belong to antimicrobial classes also applied to treat infections in humans. This molecular similarity may drive the development and selection of mechanisms resulting in cross-resistance, which justifies the necessity of integrated actions to control the evolution and dissemination of antimicrobial resistance [21]. 

Due to the potential influence of veterinary medicines in human health, Brazilian regulatory authorities establish the Acceptable Daily Intake (ADI) and the Maximum Residue Limit (MRL) of veterinary medicines in food, including antimicrobial agents, frequently based on Codex Alimentarius standards. Thus, the competence to determine the ADI and MRL values belong to the Brazilian national health surveillance agency (ANVISA), while the Ministry of Agriculture, Livestock and Supply (MAPA) is responsible for the registration and supervision of veterinary products [22].

In addition, national control programs/plans regarding antibiotics were established in previous years, among which, four stand out. The Program of Analysis of Residues of Veterinary Medicines in Animal Foods—PAMVET (from the Portuguese designation “Programa de Análise de Resíduos de Medicamentos Veterinários em Alimentos de Origem Animal”) monitored the use veterinary medicinal products in food-producing animals from 2002 to 2007. The National Program for Monitoring Prevalence and Bacterial Resistance in Chicken—PREBAF (from the Portuguese designation “Programa Nacional de Monitoramento da Prevalência e da Resistência Bacteriana em Frangos”) evaluated the prevalence and the antimicrobial resistance profile of *Salmonella* spp. and *Enterococcus* spp. in chicken meat marketed in Brazil from 2004 to 2006. The National Plan for Control of Waste and Contaminants—PNCRC/Animal (from the Portuguese designation “Plano Nacional de Controle de Resíduos e Contaminantes”) has evaluated, since 2010, the presence of residues and contaminants in eggs, milk, and honey sent for processing, as well as animals sent for slaughter, in establishments under federal inspection. The Sanitary Surveillance Action Plan on Antimicrobial Resistance has been promoting actions for the prevention and control of antimicrobial resistance in the country, articulated between human health, animal health, and agriculture, since 2015. Brazil, as a signatory member, has been participating (since 2015) in the Food and Agriculture Organization (FAO)–WHO–the World Organization for Animal Health (OIE) global monitoring system, which is a self-assessment system for reviewing and summarizing countries’ progress with multisectoral work on antimicrobial resistance.

Results of programs focused on residues of antimicrobial agents on food indicated overall scarce contamination [23,24]. In contrast, the survey performed by PREBAF showed a low prevalence of *Salmonella* spp., but a high incidence of multidrug resistance, especially in *Salmonella* Enteritidis and *Salmonella* Heidelberg serotypes. *Enterococcus* spp. was isolated from almost 100% of the samples, and resistance to streptomycin, tetracycline, erythromycin, ciprofloxacin, and chloramphenicol was often detected [25].

Regarding the FAO-WHO-OIE global monitoring system, in the most recent report (with information about the status of countries concerning the implementation of the global action plan, and actions to combat antimicrobial resistance in all sectors), Brazil is classified at level 2, with an action plan still under development. Meanwhile, other countries, such as China and the USA, are at level 5, characterized by having an action plan in progress, with an ongoing monitoring and evaluation process [26].

The FAO believes that in the coming years, there will be pressure for livestock to meet the growing demand for food, especially in the BRICS countries (Brazil, Russia, Indian, China, and South Africa). This scenario could increase antimicrobial use to ensure productivity and animal health in search of new global markets, which may increase the spread of antimicrobial resistance (AMR) in terms of their prevalence and diversity of resistance genes [27]. It also considers that medium and small family-run farms (mainly pig and poultry farms) are less monitored and regulated for antimicrobial use than intensive systems. Thus, there is a potential risk of AMR in these systems, which can spread through improperly disposed manure and sludge (such as in nearby watercourses), or even by selling animals and their products through network marketing and marketing distribution, which can also reach international markets.

This current and future perspective makes the legislation of each country, and the role of control, monitoring, and enforcement institutions, highly relevant in achieving broad compliance to practices dedicated to reducing the risk of emergence and spread of AMR in exporting countries, influencing the threat of international spread.

## 3. Antimicrobial Resistance in Poultry Farming

Poultry production has traditionally been highly productive due to intensive husbandry practices. Brazil is the second-largest producer and the world’s largest exporter of poultry meat; thus, occupying a prominent position [28]. However, the growing challenges in the fight against multidrug-resistant (MDR) bacteria and the expansion of this resistance have led to greater control and changes in some traditional poultry practices. It also justifies more intensive trade restrictions from importing countries that generally have more restrictive conditions when it comes to using antimicrobials in animals.

Due to their greater importance and occurrence in poultry, most antimicrobial resistance data on antimicrobial resistance in isolates obtained from poultry produced in Brazil refer to three main microorganisms: *Salmonella* sp., *E. coli*, and *Campylobacter* spp. (Table 1 and Figure A1).

### 3.1. Salmonella *sp.*

In general, previous data on the resistance profile of *Salmonella* strains isolated from poultry in Brazil indicate resistance against ampicillin, some first and second-generation cephalosporins, sulfonamides, tetracycline, and nitrofurantoin. However, in the early 2000s, quinolone resistance was a rare profile in these strains [29,30]. Similar patterns were also described in some studies from the South region, with strains of serotypes Enteritidis and Hadar [30,31,32,33], and the Northeast region [34,35]. On the other hand, Penha-Filho et al. (2009) studied *Salmonella* sp. strains from São Paulo State (SE) and Goias State (MW), and described a different resistance profile, with higher resistant frequencies to quinolones, in comparison with those detected to tetracycline and sulfonamides [36].

Many studies were published in the last decade describing the antimicrobial resistance profile of *Salmonella* sp. in Brazil, most of them from the South region. There is considerable variation in the susceptibility patterns, but in general, it is possible to notice an increase in quinolone resistance over the years. Additionally, a few studies reported strains resistant to colistin and polymyxin B. Besides that, the maintenance of high levels of resistance to sulphonamides, but often susceptibility to the association of this drug with trimethoprim, remains frequently described. Nitrofurantoin resistance frequencies vary widely, some exceeding 50% and others below 10%. The same occurs with tetracycline; resistance frequencies range between 10% and 30% [35,37,38,39,40,41,42,43,44,45]. 

Quinolone resistance has been mainly reported concerning nalidixic acid, reaching values above 70%. Otherwise, resistance to fluoroquinolones is lower, including some studies reporting full susceptibility. Due to its more frequent and exclusive use in animals, higher resistance values in this group are usually found for enrofloxacin. However, very high rates to different fluoroquinolones were described in a study with *Salmonella* Gallinarum (a rare serotype) isolated from outbreaks of fowl typhoid (>80%) [45], and in another study with *S.* Enteritidis from different foodborne outbreaks (>40%) [44].

Epidemiologically important non-typhoidal *Salmonella* (NTS) serotypes linked with a high burden of foodborne *Salmonella* outbreaks in humans worldwide include Enteritidis, Typhimurium, Heidelberg, and Newport [46]. Several studies in Brazil report results of *S.* Enteritidis, which is generally the most frequently found serotype in poultry. Vaz et al. (2010), for instance, investigated the antimicrobial resistance of 96 *Salmonella* Enteritidis strains from 1995 to 2003 in the South region, a period when chloramphenicol, penicillins, tetracyclines, and sulfonamides have already been banned as growth promoters in food-producing animals. In this study, *Salmonella* Enteritidis strains were isolated from salmonellosis outbreaks (43) and poultry-related products (53). Although 43.7% of strains were sensitive to all drugs tested, resistance to sulfonamide (34.4%), trimethoprim-sulfamethoxazole (25.0%), nalidixic acid (14.6%), streptomycin (2.1%), gentamicin, and tetracycline (1.0%) was identified. All strains were susceptible to ampicillin, cefaclor, ceftazidime, ciprofloxacin, and chloramphenicol, which have been some of the antimicrobial agents of choice for human therapy in *Salmonella* sp. infections over the years [37]. Another study with 148 *Salmonella* Enteritidis strains belonging to the SE86 clonal group isolated from chicken and food related to foodborne disease in the South region showed resistance frequencies of 41.9% and 17.6% to ciprofloxacin and enrofloxacin, respectively [44]. 

Notwithstanding the epidemiological relevance of *S.* Enteritidis, in some studies in Brazil, other serovars prevailed, such as Heidelberg [42] and Senftenberg [47]. *Salmonella* Heidelberg is one of the most widely distributed serotypes worldwide, frequently associated with human diseases. This serotype also presents high resistance frequencies to ceftiofur and reduced susceptibility to a related antimicrobial agent, ceftriaxone, which could limit the options for treatment of extra-intestinal infections, since it appears to be more invasive in humans than other NTS serotypes [48,49]. In Brazil, *S.* Heidelberg has been described in chickens, especially in recent years [42,50]. Voss-Rech et al. (2019), in a study in broiler farms of South Region, reported that *S.* Heidelberg comprises the vast majority (87.5%) of serotypes isolated. Another relevant feature of this serovar is its persistence in the environment. The authors demonstrated that *S*. Heidelberg could persist in the recycled broiler litter, remaining capable of colonizing the subsequently housed broilers [51].

A national surveillance program with a focus on the resistance of bacteria isolated from chicken meat (PREBAF, performed from 2004 to 2006 with 2679 carcasses collected from all geographic regions of the country), reported a low prevalence of *Salmonella* (2.7%), but more than 50% of the isolates were MDR. A high percentage of the isolates were resistant to streptomycin (89.2%), sulfonamides (72.4%), florfenicol (59.2%), and ampicillin (44.8%). The most frequently occurring serotype was *S.* Enteritidis (48.8%). Isolates from serotype Heidelberg were resistant to ceftriaxone (75.0%) and ceftiofur (43.8%) [38].

A subsequent and similar study, which evaluated 1234 *Salmonella* sp. strains isolated between 2007 and 2011 by public and private laboratories from commercial poultry carcasses and poultry by-products from different regions of Brazil, reported a lower frequency of MDR strains (16.4%). Most of the isolates belonged to the serotypes Enteritidis, Minnesota, Typhimurium, Schwarzengrund, and Mbandaka. However, the occurrence of serotype Enteritidis decreased throughout the study from 49% in 2007 to 7.8% in 2011. The authors also reported a slight increase in resistance frequencies to ampicillin, tetracycline, and gentamicin and a marked decrease in resistance frequencies to nitrofurans (61.9 to 9.2%), nalidixic acid (44.4 to 15.5%), and folate inhibitors (11.7% to 7.2%). A small number of isolates were resistant to fluoroquinolones (0.3% of the strains since 2009) [52]. This study is probably one of the most significant ones about antimicrobial resistance of *Salmonella* in chicken meat during these years, given the size and the homogeneity of the collection evaluated. 

More recently, a meta-analysis of 29 articles published with data from *Salmonella* in Brazil between 1995 and 2014, including 2119 nontyphoidal Salmonella isolates (1272 recovered from poultry and 847 from humans), reported that the highest levels of antimicrobial resistance from isolates from poultry were verified for sulfonamides (44.3%), nalidixic acid (42.5%), and tetracycline (35.5%). Isolates from human origin were resistant mainly for sulfonamides (46.4%), tetracycline (36.9%), and ampicillin (23.6%) [53].

Nevertheless, studies performed with more restricted collections may present particular resistance patters. The analysis of 82 *Salmonella* sp. isolates recovered from drag swabs between 2009 and 2010 in commercial broiler farms from Santa Catarina (S), Paraná (S), and Mato Grosso do Sul (MW) demonstrated that the higher resistance frequencies were to tetracycline (52.4%); streptomycin (24.4%); trimethoprim with sulfamethoxazole (17.1%), and ceftiofur (12.2%). All isolates were susceptible to fluoroquinolones [50]. Considering isolates recovered from chicken carcasses obtained in Mato Grosso State (MW) during 2014−2015, the analysis of resistance profile and serotypes of *Salmonella* sp. revealed 12.9% MDR strains; nearly all isolates were resistant to folate pathway inhibitors but susceptible to florfenicol, streptomycin, nalidixic acid, ciprofloxacin, enrofloxacin, and nitrofurantoin. Over 70% of the isolates belonged to serotypes *Salmonella* Infantis, *Salmonella* Abony, and *Salmonella* Agona [54]. In contrast, among 98 different *Salmonella* sp. isolates obtained from 300 frozen cuts of chicken collected in Paraná State (S) in 2015 and 2016, high resistance frequencies were observed for nalidixic acid (95%), tetracycline (94%), doxycycline (94%), ampicillin (87%), amoxicillin with clavulanic acid (84%), ceftriaxone (79%), and ciprofloxacin (76%). More than 80% of the isolates were MDR, of which 13 isolates encoded beta-lactamase genes, especially *bla*_CTX-M-2_ e *bla*_TEM-1_. The major serotypes identified were *Salmonella* Typhimurium (43%) and *S.* Heidelberg (39%) [55]. 

In a recent study, the resistance profile of 163 *Salmonella* sp. strains from 11 serotypes was compared, and some differences according to the serotypes were observed. *S.* Typhimurium, *S*. Bredeney, *Salmonella* Schwarzengrund, and *Salmonella* Tennessee had the highest overall resistance frequencies. However, this result could be influenced by the limited number of isolates of the three last serotypes. Isolates of serotypes Enteritidis, (n = 70) and Heidelberg (n = 49) were less resistant, including to ceftiofur [56]. With an alternative approach, another recently published study evaluated 264 *Salmonella* sp. strains recovered from poultry and swine isolated between 2000 and 2016 by whole genome sequencing, revealing the occurrence and persistence of international lineages of serotypes with multidrug resistance and virulent background [57].

### 3.2. E. coli

When compared with *Salmonella* sp., *E. coli* strains have higher resistance frequencies, but generally they are resistant to the same antimicrobial agents. Moreover, no significant differences in resistance levels have been detected for commensal isolates, extraintestinal pathogenic *E. coli* (ExPEC), or Avian Pathogenic *E. coli* (APEC) strains.

Regarding beta-lactams, ampicillin resistance frequency varies from around 20% [58] to over 80% [59], reaching 100% in clinical isolates [60,61]. Similar data have been described for first-generation cephalosporins [62,63], while low resistance has been reported to second and third-generation cephalosporins [64], except for ceftiofur, despite reports of low resistance frequencies in some collections [62,65]. Resistance to tetracycline is also reported with high frequencies, usually over 70%, but with exceptions again, including 13.3% in APEC strains [61]. The same study reported a low resistance frequency to enrofloxacin (6.7%). It is also important to highlight the high resistance percentage against quinolones, not only to nalidixic acid but also with fluoroquinolones, including enrofloxacin [63,65,66]. However, some opposite data show the absence of resistance to quinolones in isolates from avian-derived organic fertilizers [58]. Low resistance frequency to sulphamethoxazole-trimethoprim was also reported in this study, contrasting with most data [59,60,61,66]. Among aminoglycosides, high resistance prevalence to streptomycin is widespread, but not to other drugs of this class [61,62,63,67]. Colistin and polymyxin B resistance were rarely described [59].

Furthermore, multidrug resistance has been frequently reported. The majority of strains isolated from commercial poultry of Pernambuco State (NE) were MDR (33/35, 94%) [68]. In a study conducted in Bahia State (NE), the majority of ExPEC isolates were resistant to at least four antimicrobial agents from different classes. The most common resistance phenotypes were observed to levofloxacin (51.8%), amoxicillin/clavulanic acid (70.4%), ampicillin (81.5%), cephalothin (88.8%), tetracycline (100%), and streptomycin (100%). The overall multidrug resistance varied from 4 to 11 antimicrobials and reached 92.6% of *E. coli* strains. In addition, 40.7% of the strains were simultaneously resistant to streptomycin, levofloxacin, ciprofloxacin, and tetracycline. The proportion of highly multidrug-resistant strains (8–11 antimicrobial agents) reached 22.2%. Conversely, the aminoglycoside amikacin of avian and human use was very effective against 89.9% of ExPEC [63].

Another study compared the antimicrobial susceptibility profile of *E. coli* strains isolated from free-range and conventional raising animals. Strains from conventionally raised chickens had a higher frequency of antimicrobial resistance for the 15 antibiotics tested, as well as exhibited genes encoding extended-spectrum β-lactamase (ESBL) and ampicillin C (AmpC), unlike free-range poultry isolates. The frequency of antimicrobial resistance in strains from free-range poultry was low, except for tetracycline (60%), whereas isolates from conventional poultry showed high resistance frequencies, mainly to tetracycline, nalidixic acid, and ampicillin [69].

Carvalho et al. (2015) described the susceptibility profile of 109 *E. coli* strains isolated from the soil of broiler houses from the Rio Grande do Sul State (S). All but two isolates were resistant to, at least, one of the antimicrobial agents. More than 75% of the isolates were resistant to the tetracycline and quinolone classes. Overall, multidrug resistance patterns were found in approximately 91% of the *E. coli* isolates [66]. Braga et al. (2016) in Minas Gerais State (SE) reported the antibiotic resistance profile of 15 *E. coli* strains isolated from 2012 to 2014 from bone lesions showing a high proportion of MDR strains (73%), mainly to quinolones and beta-lactams, including third-generation cephalosporin. The percentage of resistance to tetracycline was moderate (33%), but always associated with multidrug resistance [65]. Vaz et al. (2017) in Pernambuco State (NE) studied *E. coli* strains isolated from poultry liver carcasses and detected multidrug resistance with frequencies up to 48%, varying according to the farm [67]. Borzi et al. (2017) studied *E. coli* recovered from free-range helmeted guinea fowl in São Paulo State (SE), and multidrug resistance was detected in 90.4% of the isolates [62]. Moreover, Maciel et al. (2017) reported that two *E. coli* isolates recovered from an avian colisepticemia outbreak in the Rio Grande do Sul State (S) had resistance to all antimicrobial agents tested (ampicillin, tetracycline, gentamicin, neomycin, sulfa-trimethoprim, enrofloxacin, and norfloxacin) [60].

### 3.3. Campylobacter *spp*.

Despite the often high occurrence of *Campylobacter* spp. in poultry and its importance in human disease, studies regarding the resistance profile within this genus are not common, which could be explained by the difficulty of cultivating, isolating, and maintaining these bacteria in the laboratory. However, some previous data pointed to high resistance frequencies [70,71]. The data available in the last decade demonstrate the maintenance of these profiles. 

A study from Korea with 173 *Campylobacter* spp. strains isolated between 2004 and 2008, including 27 isolates recovered from chicken meat samples imported from Brazil, reported a high frequency (80.9%) of MDR strains. Simultaneous resistance to ciprofloxacin, nalidixic acid, ampicillin, and tetracycline was the most frequent phenotype among Brazilian strains. Resistance to all drugs tested was found in the isolates originating from Brazil, except for florfenicol [72]. 

Similar results were found in the federal district (MW), with 16 *Campylobacter jejuni* strains isolated from chicken carcasses with high resistance to ciprofloxacin (100%), nalidixic acid, streptomycin, tetracycline, gentamycin (94% each), and chloramphenicol (38%), and in Parana State (S) in a study with *C. jejuni, C. coli,* and *C. lari* with detection of 75% of MDR strains, and high frequencies of resistance (>70%) to cephalothin, nalidixic acid, ciprofloxacin, and tetracycline [64,70].

More recently, Melo et al. (2019) evaluated the antimicrobial resistance of 99 *C. jejuni* isolated from chilled chicken carcasses collected in slaughterhouses in Minas Gerais State (SE) during two distinct periods (2011–2012 and 2015–2016). The prevalence of *C. jejuni* was significantly reduced in 2015–2016, as well as the number of the drug (and multidrug) resistant isolates, except for tetracycline. During the studied period, stricter regulations to control pathogens in poultry farms and slaughterhouses were implemented in Brazil, which may have contributed to the profile variation observed due to changes in selective pressures on bacterial populations [73]. 

## 4. Antimicrobial Resistance in Pig Breeding

Brazil is one of the largest animal protein producers, with 3.75 million tons of pork meat produced in 2017, where 697,000 tons were exported to more than 70 different countries. Santa Catarina State (S) accounted for more than 40% of all exported Brazilian pork meat [28]. 

Antimicrobial resistance in pig breeding is of high relevance. However, while more than 2000 articles on this subject have been published in the last ten years across the globe, just over 20 are from Brazil, highlighting the need for more local studies. Most studies focus on antimicrobial resistance and identification of worldwide clones of Gram-negative bacteria, with very few data regarding Gram-positive genus available. High frequencies of antimicrobial resistance could be found among strains obtained from pigs in different moments of the production chain (Table 2 and Figure A1). 

### 4.1. Salmonella *spp*.

Nontyphoidal *Salmonella enterica* is a common cause of foodborne disease outbreaks in Brazil and other countries [77,78]. Pigs can often become asymptomatic carriers of *Salmonella*, increasing the probability of food product contamination during slaughter and processing [79]. The widespread use of antibiotics in different steps of swine production can favor the emergence of MDR strains, which is also facilitated by mobile elements [80]. In Brazil, previous studies reported a prevalence of 24% of *Salmonella* prevalence in pork carcasses, and a high frequency of antimicrobial resistance was described in strains isolated from pork production [80,81].

The emergence of quinolone resistance is of particular concern because ciprofloxacin is a vital drug to treat serious *Salmonella* infections [82]. In the early 2010s, ciprofloxacin resistance among *Salmonella* sp. isolates was not evaluate or was lower than 4% [80,81,83,84]. In contrast, in 2019, Viana et al. identified up to 50% of resistance to ciprofloxacin among 112 isolates obtained from pig lots that have undergone prophylaxis with ciprofloxacin during growing and finishing production steps [85]. Regarding nalidixic acid, studies report rising rates over the years, from 5% in 2011 [73] to values varying from 30% to 60% in 2015 and 2016 [80,83,84]. 

Among *Salmonella* sp. of swine-origin, resistance to tetracycline seems to be frequent. In 2011, a high frequency of resistance to this antimicrobial agent was already reported (79%) [81]. Those high frequencies were also observed throughout the decade, with values in different collections of isolates, such as 54.5% in 2015 [80], 97.4% in 2017 [84], and 88.1% in 2019 [85]. Resistance to beta-lactams was also commonly found. Studies published in 2011 and 2015 pointed to ampicillin resistance frequencies of 29% and 46% [80,81]. However, the few studies released in the following years with Brazilian isolates suggest a rising rate of resistance to ampicillin and amoxicillin, with reports as high as 80% to 90% of the isolates in studies published in 2017 and 2019 [84,85]. That trend was not observed for cephalosporins resistance with low prevalence hitherto described [81,85].

Resistance frequencies to folate inhibitors are generally high but variable, similar to the strains isolated from poultry [35,37,38,40,42,52,80,81,83,84]. However, the frequency of aminoglycoside resistance frequencies is generally higher in *Salmonella* strains isolated from pigs (Table 1 and Table 2) [38,41,52,75,81,84,85]. 

Despite the high diversity of serotypes reported, the *Salmonella* serotypes more frequently recovered from pigs in Brazil are *S.* Typhimurium and *Salmonella* Derby [80,81,83]. 

### 4.2. E. coli

Urinary tract infection (UTI) is a major cause of mortality and reduced life of sows. In a study with isolates recovered from urine samples of sows with clinical signs raised in São Paulo State (SE), Spindola et al. (2018) observed that 98% of *E. coli* isolates were MDR. The authors reported resistance to ampicillin in 80% of the 186 strains tested, while resistance to amoxicillin/clavulanic acid was observed in only two strains (1.1%), and resistance to cefoxitin and ceftiofur ranged from 1.1% to 2.6%. Resistance to cefotaxime was not detected. More than 80% of these isolates were resistant to sulfonamides (94.6%), tetracycline (91.9%), and florfenicol (83.3%); 50% to 70% of the isolates were reported as resistant to nalidixic acid (66.1%), sulfamethoxazole-trimethoprim (59.6%) and streptomycin (52.6%). Resistance frequencies lower than 25% were detected to ciprofloxacin (22.5%), spectinomycin (11.2%), and gentamycin (2.6%) [86]. In contrast, Silva et al. reported eight isolates obtained in 2012 also in the same state from pig fecal swabs resistant to third- and fourth-generation cephalosporins, associated with the occurrence of the ESBL encoding gene *bla*_CTX-M-15_. These isolates were also resistant to ciprofloxacin, enrofloxacin, norfloxacin, tetracycline, sulfonamide-trimethoprim, and gentamicin, being sensitive only to amikacin, cephamycins, and carbapenems [87].

In veterinary medicine, colistin sulfate is mainly used in oral preparations, due to its excellent activity against *E. coli* and *S. enterica*, low frequency of resistance, and poor absorption after oral administration, especially in pigs and poultry production. However, in the last few years, colistin-resistant *E. coli* is becoming more common. In 2012, resistance to colistin could already be observed in *E. coli* isolated from pigs. Morales et al. (2012) used the agar dilution test, which was then considered the gold standard for colistin susceptibility evaluation, and observed that 6.3% of *E. coli* isolates were resistant to colistin [88]. Recently, Kiefer et al. (2018) evaluated 126 pig samples and identified eight colistin-resistant *E. coli* isolates. Among them, a single isolate was positive by PCR for the *mcr* gene. This isolate was also resistant to broad-spectrum cephalosporins, tetracycline, chloramphenicol, florfenicol, nalidixic acid, sulfonamides, sulfamethoxazole-trimethoprim, and kanamycin [89].

### 4.3. Yersinia enterocolitica

Pigs are considered natural reservoirs of *Y. enterocolitica*, which explains its presence in slaughterhouses, and the association between pork consumption and yersiniosis. As this pathogen persists in the pork chain from the initial steps of production, contamination of carcasses and pork products can occur, particularly during handling of the head, tongue, and palatine tonsils [90]. In addition, *Y. enterocolitica* may be present in the intestinal contents and mesenteric lymph nodes of pigs, which are also considered relevant sources of contamination during slaughtering [91]. 

Only a few studies regarding this agent in Brazil have been conducted. In the past ten years, the occurrence of *Y. enterocolitica* was low, but still suggesting that pigs serve as a primary source in the transmission of this bacteria to humans [92]. Noteworthy, multidrug resistance is commonly found among isolates of this species in Brazil, with variable resistance profiles [91,92,93]. 

Among these isolates, resistance to beta-lactams was widespread. Frazão et al. (2017) studied 34 isolates obtained in 30 years, and most of them (94.1%) were resistant to ampicillin and ticarcillin even when associated with a beta-lactamase inhibitor, the resistance persisted [93]. Other studies could also observe high frequencies of resistance to aminopenicillins [91,92]. Among 16 isolates obtained from 10 pig production lots, this trend was also described to other beta-lactams, such as cephalosporins and carbapenems, both in frequencies as high as 100% [91]. 

Resistance to fluoroquinolones was very unusual, with only one isolate from all Brazilian studies displaying this phenotype. Resistance to nalidixic acid (NAL) was more common but not frequent. Indeed, other studies demonstrated that frequencies of fluoroquinolone resistance were significantly lower than those observed for nalidixic acid alone [94]. 

## 5. Antimicrobial Resistance in Dairy and Beef Cattle Breeding

### 5.1. Staphylococcus aureus and other Staphylococcus *spp*.

Brazil is the world’s largest milk producer, accounting for 7% of milk produced in the world. Minas Gerais State (SE) is the largest producer, followed by the Rio Grande do Sul (S), Paraná (S), Goiás (MW), Santa Catarina (S), São Paulo (SE), and Bahia (NE) States [96]. Bovine mastitis is an infectious disease that impacts milk production, leading to economic loss and public health concern. It may present as subclinical, clinical (or acute), and chronic forms, with subclinical mastitis being the most common. Since clinical mastitis may be associated with higher virulence and antimicrobial resistance profiles of the microorganism, this information is presented in Table 3 and along with the text, when available and necessary. According to the National Syndicate of the Animal Health Products Industry, there are 166 veterinary products to treat mastitis marketed in Brazil, including beta-lactams, macrolides, tetracycline, quinolones, sulfonamides, and others [97]. 

Although different species may cause mastitis, *S. aureus* is one of the most frequently isolated etiological agents of these infections, which justifies the largest number of studies regarding this species [98]. Most published data are from *S. aureus* isolates recovered from herds localized in the Southeast and South states. In general, the comparison of resistance frequencies as a function of herd location has limitations, mainly because studies that include isolates from different states sometimes do not discriminate the origin of the samples. Similarly, data on the sample collection period have not been reported in several studies, which does not allow reliable temporal analysis. Table 3 presents the available data on *Staphylococcus* spp. mastitis resistance profile (see also Figure A1), and Table 4 shows the genes detected in these isolates.

Susceptibility to the majority or all antimicrobial agents tested against *S. aureus* isolates from bovine mastitis has been observed in Brazilian herds [90,91,92,99,100,101]. However, resistance frequencies have increased for some antimicrobial agents compared to studies published in earlier periods [102,103,104,105].

Beta-lactam resistance, especially to penicillinase-labile penicillins (PSLP; for example, amoxicillin, ampicillin, penicillin G), has been widely observed among *Staphylococcus* isolates recovered from bovine mastitis [91,97,98,99,100,106,107,108]. Penicillin G resistance frequencies have ranged from 30.4% to 100% in the studies published in the last ten years [100,101,109,110]. Some studies have also tested other PSLPs, sometimes with slightly higher resistance frequencies than penicillin G [106,111,112,113]. 

Marques et al. (2017) detected the *bla*Z gene in 14 of 20 isolates considered resistant to penicillin G by the edge zone, and among them, only five isolates had the phenotype confirmed by disk diffusion test [110]. High PSLP resistance frequencies (>80%) were observed in isolates obtained between 2004 and 2008 from cows with clinical (59 isolates) and subclinical (293 isolates) mastitis belonging to 38 herds [114]. In another study in Minas Gerais State (SE), 266 *S. aureus* isolates were characterized to assess the susceptibility to ampicillin, penicillin G, and tetracycline. Resistance to ampicillin and penicillin G was detected in 66.5% and 70.7% of the isolates, respectively. Ninety isolates resistant to the antimicrobial agents originally tested were subjected to minimum inhibitory concentration (MIC) and investigation of resistance genes. The MIC_50_ and MIC_90_ were, respectively, 1 µg/mL and 2 µg/mL for ampicillin and 0.5 µg/mL and 1 µg/mL for penicillin. The *bla*Z gene was detected in almost all isolates [108]. Another study evaluated the antimicrobial susceptibility profile of 46 bacteriocin-producing *S. aureus* isolates obtained from cows with mastitis from 12 herds. Resistance to PSLP was prevalent among these isolates, being 67.4% and 65.2% of the isolates resistant to ampicillin and penicillin G, respectively [106].

The PSLP resistance frequencies were higher among *S. aureus* isolates recovered from lactating cows than from heifers in two experimental herds in São Paulo State (SE). Eighty-three and 27 isolates of heifers and cows, respectively, were evaluated. Among heifer isolates, 39.6% and 14.5% were resistant to penicillin G and ampicillin, respectively. In the case of cow isolates, 62.9% and 40.7% were resistant to penicillin G and ampicillin, respectively [111].

In some studies, PSLP resistance was not prevalent compared to other antimicrobial agents, but significant frequencies were observed. Among 27 *S. aureus* and three coagulase-negative *Staphylococcus* (CNS) isolates characterized by Freitas et al. (2018), the highest resistance frequencies were observed for trimethoprim (100%), neomycin and tetracycline (96.7% each). However, 70% of the isolates were resistant to penicillin G [113]. Haubert et al. (2017) reported a penicillin G resistance frequency of 48% among *S. aureus* isolates, but the resistance rate to sulfonamides was higher (65%) [112]. 

Few studies have been published on the antimicrobial susceptibility profiles of CNS and coagulase-positive staphylococci other than *S. aureus* (oCPS) from bovine mastitis. Fernandes dos Santos et al. (2016) characterized isolates obtained between 2008 and 2010, in three different regions of the country and identified 79 of 1365 *S. aureus*, and 91 of 1484 CNS displaying smaller growth inhibition zones for oxacillin. Considering these sub-groups, a penicillin G resistance frequency of 30.4% and 34.1% for *S. aureus* and CNS were detected, respectively [109]. Laport et al. (2012) observed penicillin G resistance among 51% of CNS isolates of an older collection (1995–2003) obtained from bovine mastitis in the Southeast region [107]. Da Costa Krewer et al. (2015) evaluated the antimicrobial resistance profiles of 126 *S. aureus,* 61 oCPS, and 31 CNS isolates obtained of herds from the Northeast region. The total PSLP resistance frequency was higher than 60%, and the lowest frequency (36%) was observed for CNS [100].

Penicillinase-stable penicillins (PSSP), of which methicillin is a prototype, are semi-synthetic drugs that have been developed to treat infections caused by beta-lactamase-producing *S. aureus*. Methicillin-resistant *S. aureus* strains (MRSA) have resistance to all beta-lactam agents, except for new fifth generation cephalosporins. The gene most commonly related to methicillin resistance is the *mec*A, but a *mec*A homolog gene (*mec*C), still rarely detected, has been described [115,116]. 

Cloxacillin is one PSSP that may be used to treat bovine mastitis and, consequently, may contribute to the selection of strains resistant to this antimicrobial group. MRSA isolation has been reported from mastitis cases in Brazilian herds, but the prevalence is low. In most studies, MRSA strains were not isolated [100,106,109,110,111] or were isolated from few animals [101,114,117,118]. In 2012, Costa et al. published a study with milk samples from 38 herds, in which 2% of *S. aureus* isolates from mastitis were MRSA [114]. A similar MRSA isolation prevalence (1.4%) was observed by Bonsaglia et al. (2018), who analyzed 285 isolates recovered from 18 herds in São Paulo State (SE). They detected neither the *mecA* nor the *mecC* genes among the isolates [101]. Guimarães et al. (2017) reported an outbreak of MRSA intramammary infections in Dutch cows from a dairy herd in São Paulo (SE). From a total of 103 cows investigated, 12.2% of the mastitis cases were caused by *S. aureus*. In this collection of isolates, *mecA* was also detected in isolates that did not express the resistance phenotype (OS-MRSA—oxacillin-susceptible *mec*A-positive *S. aureus*) [119]. Haubert et al. (2017) reported MRSA isolation, identified by phenotypic methods, in herds from the Rio Grande do Sul State (S). However, the authors did not detect the *mec*A gene among the isolates [112].

Some studies have also investigated the methicillin resistance among isolates of other *Staphylococcus* species. Approximately 18% of 82 *S. aureus* isolates and 99 other staphylococci obtained from herds from six states exhibited resistance to methicillin by epsilometer test but only one strain was MRSA. Methicillin-resistance was also observed among isolates of *Staphylococcus chromogenes, Staphylococcus epidermidis, Staphylococcus haemolyticus, Staphylococcus saprophyticus, Staphylococcus simulans, Staphylococcus xylosus,* and *Staphylococcus warneri*, but the gene *mec*A gene was detected only in eight *S. epidermidis* isolates [118]. Besides, 26 methicillin-resistant *Staphylococcus* (MRS) isolates belonging to the species *S. epidermidis*, *S. chromogenes*, *S. warneri*, *S. simulans,* and *Staphylococcus hyicus* were recovered from CMT-positive cows from 11 herds in São Paulo (SE), all of them phenotypically resistant to PSSP and carrying the gene *mecA* [117]. In another study, methicillin non-susceptible CNS isolates were obtained from dairy herds localized in Southern and Southeastern States. Nine of them, all *S. epidermidis*, carried the *mec*A gene, and two *Staphylococcus sciuri* isolates had a *mecA-*homolog gene. The *mec*C gene was screened but not detected in any of the isolates [109]. Furthermore, some studies identified low frequencies (<5%) of MRS isolation among strains of cows with mastitis [100,107]. 

Resistance frequencies for tetracycline have varied in different studies published over the period evaluated [106,108,113,114]. The highest rate reported (96.7%) was observed among isolates obtained in herds from the Rio Grande do Sul (S). In this study, the isolates also presented high resistance frequencies to other antimicrobial agents [113]. In the same State, a lower frequency of resistance to tetracycline (39%) was observed. Sulfonamide resistance frequency (65%) was the highest among the drugs tested [112]. Low tetracycline resistance frequencies (<10%) have also been reported among *S. aureus* isolates [101,109,110,117]. In most of these studies, the resistance frequencies to other antimicrobial agents tested were low as well [101,117]. In a study performed in the Northeast region with 195 isolates of different staphylococcal species, 17.4% and 11.9 % of the isolates were resistant to tetracycline and doxycycline, respectively [100]. Laport et al. (2012) reported a tetracycline resistance rate of 14.3% among 54 CNS isolates recovered from herds located in the Southeast region [107]. In another study, the authors observed higher tetracycline resistance frequencies among CNS (24.2%) compared to *S. aureus* (8.9%) isolates [109].

Erythromycin and clindamycin are often tested for *S. aureus* isolates recovered from bovine mastitis. Although macrolide resistance frequencies have also varied in different studies, in most of them, the frequencies were 0%–10% [101,110,117]. The highest erythromycin resistance frequency was 58.7% [106]. For lincosamides, resistance frequencies ranged from 0%–52%, most of them less than 15%. Among CNS, the highest resistance frequency (18.4%) was reported by Laport et al. (2015). The authors also reported resistance to clindamycin, but at a lower prevalence [101,107,112].

In most studies published, the quinolone resistance frequencies have been low [100,101,112]. However, high resistance frequencies have been reported in specific studies [110,113]. Freitas et al. (2018) reported 43.3% of enrofloxacin-resistant *Staphylococcus* spp. isolates. This result is questionable because interpretation criteria were not clear [113]. In another study, the resistance frequencies ranged from 20% to 25%, according to the quinolone tested [110]. 

For the treatment of bovine mastitis, aminoglycosides may also be used. Gentamycin resistance frequencies observed are generally lower when compared to other aminoglycosides, ranging from 0% to 2% [101,107,109,111], while resistance against streptomycin is higher, although only a few studies have evaluated this drug. Marques et al. (2017) and da Costa Krewer et al. (2015) reported a streptomycin resistance rate of 25% and 11.9%, respectively [100,110]. High frequencies of resistance were observed for gentamicin (86.7%) and neomycin (96.7%) in the study by Freitas et al. (2018); however, they analyzed a small number of bacterial strains and did not inform the interpretation criteria [113].

Although glycopeptides are not employed for the treatment of bovine mastitis, isolates have been evaluated for vancomycin susceptibility due to its importance to the treatment of human infections. Two studies reported the absence of vancomycin resistance through by the disk diffusion method; however, they did not employ the agar dilution method recommended for this evaluation [101,106]. On the other hand, Mello et al. (2017) detected vancomycin heteroresistance in 7.2% of the 181 isolates [118].

Trimethoprim/sulfamethoxazole resistance, also prescribed for the treatment of bovine mastitis, has not been detected or detected in less than 10% of the isolates [100,101,109,114,117]. Although sulfonamide resistance frequency has been very high in one study (65%), trimethoprim/sulfamethoxazole resistance rate was also low (3%) [112]. Regarding other antimicrobial agents, resistance to amphenicols [110,114,117] and novobiocin [110,114] also has been reported in some studies.

### 5.2. Streptococcus *spp*.

Streptococcal species, especially *Streptococcus agalactiae*, are also critical etiologic agents of bovine mastitis. However, data on resistance of these bacteria isolated from Brazilian herds are very scarce. Recently, Miranda et al. (2018) analyzed 16 *S. agalactiae* isolates from subclinical mastitis from five herds localized in Pernambuco State (NE). All the isolates were susceptible to penicillin, ceftriaxone, levofloxacin, chloramphenicol, linezolid, and vancomycin. The highest resistance frequency was observed for tetracycline (87.5%). Erythromycin and clindamycin resistant isolates were also resistant to tetracycline, being these strains considered as MDR (25%) [120]. In another study, *S. agalactiae* isolates from bovine and human were analyzed to determine their serotypes and antimicrobial susceptibility profiles. Only 29 of 392 isolates were obtained from milk samples of cows with mastitis. The bovine isolates were collected in two periods (1987–1989 and 2003–2006) from five herds localized in three Southeastern states. Resistance was observed to tetracycline (89.6%), erythromycin (27.6%), and clindamycin (20.7%), which frequencies were higher than those reported for the isolates of human origin [121].

### 5.3. E. coli, Salmonella *sp*., and Listeria monocytogenes

Even though *E. coli* is a relevant etiologic agent of environmental mastitis, scarce data on antimicrobial resistance are available. Fernandes et al. (2011) analyzed 27 *E. coli* isolates obtained from clinical mastitis recovered from seven herds localized in Minas Gerais State (SE). The isolates were resistant or intermediate to trimethoprim-sulfamethoxazole (51.8%) and ampicillin (14.8%), and some isolates were simultaneously non-susceptible to neomycin (3.7%) [122]. In another study, 260 pasteurized cow’s milk samples were collected in commercial establishments from Paraná State (S), from 2000 to 2007. Among the coliform isolates, *E. coli* was identified in 77.05% of the samples. The highest resistance frequency was observed for cephalothin (23.4%), followed by ampicillin (19.2) and tetracycline (10.6%). Resistance frequencies less than 6.5% were seen for amoxicillin-clavulanic acid, trimethoprim-sulfamethoxazole, and ciprofloxacin. None of the isolates produced ESBLs [123]. Alves et al. (2018) detected flies carrying MDR *E. coli* isolates in two dairy farms from São Paulo State (SE). Resistance was observed for ampicillin, amoxicillin-clavulanic acid, tetracycline, trimethoprim-sulfamethoxazole, enrofloxacin, chloramphenicol, and ciprofloxacin, associated with the occurrence of *bla*_TEM_ (36.3%), *tetA* (14.8%), and *bla*_CTX-M_ (11.1%) genes [124]. 

Regarding beef cattle, Brazil is the world’s largest exporter, with the second world’s largest herd of cattle (232 million) and reaching a beef production of 9.9 million tons [125]. However, in contrast to poultry and pigs, production is often extensive, reducing the necessity of antibiotic usage. Nevertheless, large consumer markets require monitoring of specific bacterial species such as *E. coli*, *Salmonella* sp., and *Listeria* sp. to ensure pathogen-free production. 

In the last ten years, some studies have evaluated antimicrobial resistance of *E. coli*, *Salmonella,* and *Listeria monocytogenes* isolates obtained from cattle carcasses, beef-products, and meat-processing environments. Moreover, dos Santos et al. (2018) investigated the contamination by Shiga toxin-producing *E. coli* (STEC) of beef and carcasses from one slaughterhouse located in Mato Grosso State (MW). All 18 STEC isolates detected were susceptible to 12 antimicrobial agents tested, except streptomycin [126]. In contrast, the MDR profile has been detected among *E. coli* isolates [127], including serotype O157: H7 isolates [128]. 

Few *Salmonella* sp. isolates have been evaluated for antimicrobial resistance due to low levels of their detection in carcasses, production line, and slaughterhouse environment [127,128,129,130]. *Salmonella* sp. isolates susceptible to all antimicrobial agents tested were observed in some studies [127,130]. However, isolates resistant to trimethoprim-sulfamethoxazole, sulfamethoxazole, and tetracycline were detected in cutting boards of butcher shops from Minas Gerais State (SE). All isolates were susceptible to amikacin and cefotaxime [129].

*Listeria monocytogenes* obtained from carcasses have also been evaluated for antimicrobial susceptibility. Susceptibility has been observed for most drugs tested [128,131]. Camargo et al. (2015) evaluated antimicrobial resistance of *L. monocytogenes* isolates recovered from meat-processing environments, beef products, and clinical cases. All the isolates were susceptible to most of the tested antimicrobial agents tested, but many strains were resistant or intermediately resistant to clindamycin (88.3%) and oxacillin (73.7%) [131].

## 6. Mobile Genetic Elements Associated with Emergent Antimicrobial Resistance Mechanisms Detected in Isolates from Farm Animals and Animal-Derived Foods Produced in Brazil 

### 6.1. β-Lactams Resistance-ESBL and Plasmid-Mediated AmpC (pAmpC)

First reports of *bla*_CTX-M-2,_ *bla*_CTX-M-8,_ and *bla*_CMY-2_ in animal-derived foods produced in Brazil date from 2008 to 2010 in studies involving chicken meat imported by Denmark and the United Kingdom [134,135]. Since then, particularly after 2014, extended-spectrum β-lactamase (ESBL) and plasmid-mediated AmpC (pAmpC) encoding-genes have been extensively reported in chicken and chicken meat produced in Brazil. Studies indicate that these genes are mainly carried by *E. coli* and nontyphoid *Salmonella,* but also by *Escherichia fergusonii, Klebsiella pneumoniae, Citrobacter diversus,* and *Proteus mirabilis* [136,137,138,139,140]. Reports include animals on farms [36,136,137,138,141,142,143,144,145,146,147], whole carcasses, or meat pieces available in Brazilian retail markets [69,136,138,140,145,148,149], and exported chicken meat [150,151,152]. The same genes were detected in turkeys with clinical signs of colibacillosis [153]. Although a study stated that the prevalence of ESBL and pAmpC genes was higher in conventional than in free-range chicken, *bla*_CTX-M-2_, *bla*_CTX-M-8_, *bla*_CTX-M-15,_ and *bla*_CMY-2_ were also detected in carcasses of antibiotic-free grown animals [69,140]. Such occurrences may be related to environmental contamination with bacteria carrying these genes. For instance, *Salmonella* sp. and *E. coli* producing *bla*_CTX-M-_variants have been detected in poultry farms environment and on the surface of flies from dairy farms, respectively [124,145]. In addition, a study has shown that the prophylactic administration of ceftiofur to one-day-old broiler chicks may be associated with early carriage of ESBL-producing *E. coli* in the gut of these animals [154]. Table 5 describes the source, geographic location of sampling, and *bla* genes found in studies that reported ESBL and pAmpC-encoding genes in food or food-producing animals in Brazil. 

In *Salmonella*, *bla*_CTX-M-2_ was identified in non-transferable and conjugative plasmids of 90 to 290 kb. These plasmids belonged to IncI1, IncHI2, or non-typable incompatibility groups [36,145,155,156]. Part of isolates characterized by Fernandes and coworkers was not able to transfer *bla*_CTX-M-2_ to *E. coli*, suggesting a possible chromosomal location of this gene [145]. In contrast, in *E. coli*, most studies suggest a chromosomal location of *bla*_CTX-M-2_ with a few reports of this gene in plasmids belonging to IncK, IncHI2, IncP incompatibility groups ranging from 35 to 280 kb [137,139,146,147,157]. Always when investigated, both in *Salmonella* sp. and in *E. coli*, this gene was surrounded by IS*CR1* and class I integron structures [36,136,137,157]. Differently, *bla*_CTX-M-8_ has been associated, both in *Salmonella* sp. and in *E. coli*, with IS10 and IncI1 conjugative plasmids ranging from 50 to 100 kb [137,138,157]. The *bla*_CTX-M-15_ gene was reported in isolates recovered in 2011 and 2014 located in IncX plasmids of 50kb [146,157]. Blast alignment suggested that an ISEcp element mobilized this gene to a plasmid previously detected in *Shigella flexneri* and *E. coli* strains [157]. 

The *bla*_CMY-2_ gene has been reported in *S.* Heidelberg and *S.* Minnesota harboring IncI plasmids varying in size from 90 to 148 kb [36,129,135]. In contrast, this genetic environment is more diverse in *E. coli*. The mobilization of the gene seems to be associated with ISEcp1, and with IncI, IncFIB, IncK, IncB/O, IncAC, and non-typable plasmids, most of them 80-90 kb long [143,146,147,157].

### 6.2. β-Lactams Resistance-mecA and Van

*Staphylococcus* β-lactam resistance mediated by the *mec*A gene has been reported in *S. aureus* (MRSA) from chicken meat, swine swabs, bovine mastitis, and cheese processing plants [99,119,158,159,160]. Also, different species of *mecA*-producing coagulase-negative strains (*S. epidermidis*, *S. chromogenes*, *S. hyicus*, *S. warneri*, *S. simulans*; methicillin-resistant CoNS (MRCoNS)) have been recovered from cows with mastitis [109,118,132]. Among *S. aureus* from mastitis or swine samples, *mecA* was associated with staphylococcal cassette chromosome *mec* (SCC*mec)* types III, IV, or V in MDR isolates [132,159,160]. Among MRCoNS, *mecA* was encoded in *SCCmec* types I, IV or V, or non-typable SCC*mec* elements, in some cases displaying only resistance to β-lactams [109,118,132]. 

In 2013 one MRSA/vancomycin-resistant *S. aureus* (VRSA) strain was isolated from chicken meat, harboring *mecA*, *vanA*, *vanB*, and *vanC2/3* genes. In this study, the same genes were detected in one *Staphylococcus intermedius* isolate. Such *van* genes were probably expressed because vancomycin minimal inhibitory concentration displayed by these isolates were 512 and 64 µg/mL, respectively [158]. To the best of our knowledge, this is the only report of VRSA in food-producing animals in Brazil. In fact, since 2005, even regarding enterococci, there are a small number of reports describing the occurrence of *van* genes in isolates from animals or animal-derived foods [161,162,163,164]. 

### 6.3. Colistin Resistance-Mcr

Colistin resistance gene determinant *mcr-1* was first described in Brazil in 2016 by screening isolates collected as part of different surveillance projects on carbapenemase and ESBL-producing bacteria. Using MacConkey agar plates supplemented with colistin (2 mg/L) followed by a polymerase chain reaction, Fernandes et al. identified *mcr-1* in *E. coli* strains from fecal samples of pigs and chickens. The samples were collected between 2012–2013 in Santa Catarina (S), Minas Gerais (SE), Paraná (S), and São Paulo (SE) states [165]. Following, *mcr-1* was identified in rectal swabs of chicken never exposed to polymyxin, collected in 2015 in the Southern region. These authors successfully transferred *mcr-1* to *E. coli* J53, demonstrating that the gene was located on plasmids [166]. The occurrence of *mcr-1* in birds not treated with polymyxin may be related to a previously undetected environmental presence of this gene [167]. For instance, a study developed with soils from a vegetable production area in Rio de Janeiro State (SE) where poultry litter is commonly used as organic fertilizer, indicated the presence of *mcr-1* not only in the production area but also, at lower concentrations, in the surrounding native vegetation [168]. 

Regarding food samples, *mcr-1* was detected in *E. coli* obtained from chicken meat samples acquired in retail markets in São Paulo State (SE), and from three of 409 chicken carcasses produced in the three Southern states, most of them in isolates carrying β-lactamase genes [149,169]. The gene has also been reported in *S.* Typhimurium, and *S.* Schwarkergund collected from pork meat, and poultry meat acquired in the Rio Grande do Sul (S) and São Paulo (SE) States, respectively [95,170]. 

In Brazil, *mcr-1* has been consistently associated with IncX4 plasmids, both in *E. coli* and *Salmonella* sp. strains, isolated from animal, animal-derived food, and clinical and environmental sources [169,170,171,172,173,174]. Moreno et al. published the complete sequence of a 32kb *mcr1*_IncX4 plasmid harbored by *Salmonella* sp. isolated from chicken meat [170]. Although hitherto, *mcr-1* is the only variant systematically identified among MCR producers in Brazil, an MCR-3-like enzyme-producing *E. coli* was recovered from swine in Minas Gerais State (SE). The isolate had colistin MIC of 4 µg/mL, and biochemical analysis demonstrated that the enzyme acts similarly to MCR-1. The *mcr-3.12* gene was located between two insertion sequences belonging to the IS*66* and IS*30* families embedded into an IncA/C_2_ plasmid [89]

### 6.4. Quinolones Resistance-Qnr 

In Brazil, the plasmid-mediated fluoroquinolones resistance *qnr* genes have been reported in samples from animals or animal-derived foods mainly after 2013. Overall, *qnrB* and *qnrS* are the most prevalent *qnr* families detected. Different *qnrB* variants (when specified recognized as *qnrB2, qnrB5*, *qnrB19*) have been reported in *E. coli*, *E. fergusonii, K oxytoca, S*. Corvallis, *S.* Schwarzengund, and *S*. Newport isolates obtained from chicken or chicken meat [36,140,147,175,176,177,178]. *qnrS* was reported in *E. coli* obtained from chicken meat and swabs from healthy poultry and pork [140,177,179]. More recently, *qnrE* was detected in an MDR *S*. Typhimurium strain isolated from a swine carcass [180]. The occurrence of *qnrA* in animal-derived samples was no reported in the studies covered by this review. 

The genetic occurrence of *qnrB* seems to be strongly associated with low molecular weight ColE plasmids [36,147,177]. Nevertheless, in *Salmonella*, the gene has also been detected in large 280 kb IncHI2A plasmids, co-located with *bla*_CTX-M-2_ [36]. The same was noticed for *qnrS*. Although Ferreira et al. described this gene in ColE plasmids, it was also described in a 49kb IncX1 plasmid carried by *E. coli*, co-located with *bla*_CTX-M-15_ [157,177]. 

## 7. Concluding Remarks 

As has been occurring around the world, there is a growing concern in Brazil regarding increased antimicrobial resistance related to farm animals. This fact is demonstrated by the number and variety of regulations that have been proposed over time, especially from 1998 onwards, when at least 14 regulations directly related to the theme have been implemented. The Brazilian official institutions of agriculture, sanitary surveillance, human health, and animal health have elaborated and executed plans and programs to control and monitor the occurrence of these microorganisms in recent years, seeking to know the Brazilian reality and establish procedures for the effective reduction of the problem. However, it is clear that a more representative, transparent, and continuing program must be done to support the position of Brazil as a producer and exporter of high-quality meat products.

The Brazilian scientific community studies different aspects of resistance to multiple antimicrobial drugs, with a significant number of publications involving microorganisms isolated from Brazilian regions that concentrate most of the meat production (beef, pork, and poultry), milk, and eggs. The data presented in this review denotes a wide variety of resistance profiles, generally irrespective of the region of the country. However, as stated above, most information comes from states or regions that concentrate the animal production, including the South region concerning poultry and pig production, and South and Southeast regions related to dairy production (Figure A1). Although there is a vast area of beef production in the North and Midwest regions, fewer data were available. However, it is important to note that cattle managing and production in Brazil are mainly extensive with no use of prophylactic or growth-enhancing antimicrobial agents, and little use of these drugs for therapeutic purposes. 

A recently published metadata study about trends in antimicrobial resistance in middle and in low-income countries, using, among other parameters, the proportion of antimicrobial agents with resistance above 50% (P50) in each study as indicators for comparisons, demonstrated that, unlike many countries in Asia, studies developed in most areas of Brazil have low P50. Some spots with P50 above 0.5 (the index is presented on a scale from 0 to 1) were reported in different regions of the country, mainly in the Southeast region, where many scientific institutions take place, or in states not classically associated with intensive animal production. However, an emerging hotspot for antimicrobial resistance was recognized in the South region of the country, which is relevant because this geographic area has a vigorous poultry and swine productive chain. Comparing the resistance profiles of cattle, chickens, and pigs in America, the analysis indicated slightly higher resistance frequencies associated with chickens than with pigs, and *E. coli* as somewhat more resistant than *Salmonella* [182]. Evaluating the resistance data presented in this review with a P50 rationale, we also identified *E. coli* as more resistant than *Salmonella* sp. in both swine and poultry. Moreover, the resistance of *Salmonella* strains isolated from pigs was higher than those obtained from poultry. Data on *Campylobacter* in poultry and *Yersinia* in pigs are scarce, but they demonstrate the high resistance of strains. Overall, these observations indicate trends; however, the analysis of a more extensive and representative set of strains is imperative to confirm these findings.

In this context, the creation of a national surveillance plan is underway in Brazil, as part of the commitments with the FAO, WHO, and OIE global initiatives. Currently, internal regulations are in place, and significant updates to growth-promoting additive control legislation are due to be implemented soon. Moreover, recently a coalition of prominent associations of livestock producers (including poultry, pig, cattle, and fish) has been formed with the mission of “promoting the responsible and rational use of antimicrobial agents, through the engagement of different links in the animal protein production chain” (https://aliancaproteinaanimal.com.br/). Brazil also has a highly capacitated and engaged scientific community. If these forces work together, a favorable perspective for the control and monitoring of antimicrobial resistance in Brazil may be provided. However, long-term investments are required, especially for research, surveillance, laboratories, human health systems, basic sanitation, animal health, and training. Political commitment, availability of financial resources, technical investments, and international collaboration can make the development and implementation of effective national plans and programs viable.

## Figures and Tables

**Table 1:** Resistance profile of *Salmonella* sp., *Escherichia coli,* and *Campylobacter* spp. isolated from poultry, Brazil (data published between 2009 and 2019).

Reference	Sampling Period	Geographic Region ^a^	Local (n)	Isolates (n)	Antimicrobial Resistance ^b^					
					Beta-lactam	Tetracycline	Quinolone	Sulfonamide	Aminoglycoside	Others
*Salmonella* sp.										
Duarte et al., 2009 [35]	2004	NE	poultry carcasses (260)	11 serotypes (19)	Amp: 10.5%	Tet: 31.6%	Cip, Eno: 5.2% Nal: 21.0% Nor: 2.5%	Sut: 5.2%	Kn: 15.8% Str: 73.7%	Clo: 5.2% Nit: 52.6%
Vaz et al., 2010 [37]	1995–2003	S	–	*S.* Enteritidis (96)	Amp, Caz: 0.0%	Tet: 1.0%	Nal: 14.6%	Sul: 34.4% Sut: 25.0%	Gen: 1.0% Str: 2.1%	–
Medeiros et al., 2011 [38]	2004–2006	N, NE, MW, SE, S	poultry carcasses (2679)	18 serotypes (250)	Amp: 38.0% Atm: 19.2% Cfl: 12.0% Cfo: 13.2% Cro: 6.0% Ctf: 28.0%	Tet: 12.0%	Cip: 4.0% Eno: 19.2% Nal: 40.0%	Sul: 58.0% Sut, Tri: 10.0%	Gen: 12.0% Str: 78.0%	Clo: 6.0% Flo: 62.0% Nit: 8.0%
Kottwitz et al., 2012 [39]	2002–2006	S	breeding chickens	*S.* Enteritidis (38)	Amp, Ctx: 0.0%	–	Cip: 0.0% Nal: 26.3%	Sut: 0.0%	–	Clo: 2.6%
Kottwitz et al., 2013 [40]	2003–2006	S	discarded hatching eggs (1000)	4 serotypes (26)	Amp, Ctx: 0.0%	–	Cip: 0.0% Nal: 23.1%	Sut: 0.0%	–	Clo: 0.0%
Costa et al., 2013 [52]	2007–2011	N, NE, MW, SE, S	broiler carcasses	61 serotypes (1234*)*	Amp: 12.4%–18.9%	Tet: 15.2–18.9%	Nal: 15.5%-44.4%	Sut: 7.2%-11.7%	Gen: 7.0–10.6%	Nit: 9.2%–61.9%
Moraes et al., 2014 [74]	–	MW	one-day-old chicks and others	12 serotypes (53)	Amp: 5.7%	Tet: 13.2%	Cip: 0.0% Eno: 5.7%	Sul: 73.6% Sut: 13.2%	Neo: 0.0%	Flo: 0.0%
Campioni et al., 2014 [41]	2004-2010	NE, MW, SE, S	–	*S.* Enteritidis (60)	Amp, Cfl, Cro: 0.0%	Tet: 0.0%	Nal: 73.3%	Sut: 0.0%	Ami, Str: 0.0%	Clo: 0.0%
Pandini et al., 2015 [42]	2010–2011	S	broiler farms (342 drag swabs)	19 serotypes (39)	Amp: 20.5% Cfl: 23.0% Imp: 0.0%	Tet: 30.8%	Cip, Nor: 0.0% Nal: 28.2%	Sut: 12.8%	Gen: 2.6% Str: 10.2% Tob: 0.0%	Clo: 2.6%
Minharro et al., 2015 [75]	2010–2011	MW, SE	poultry carcasses (300), heart (600) and livers (600)	9 serotypes (26)	Amc: 100% Amp: 0.0% Ctf: 3.8%	Dox, Tet: 0.0%	Cip: 0.0% Eno: 3.8%	Sul: 53.8% Sut: 0.0%	Gen: 3.8%	–
Voss-Rech et al., 2015 [50]	2009–2010	S, MW	broiler farms (1543 drag swabs)	15 serotypes (82)	Amc: 6.1%; Ctf: 12.2%	Tet: 55.4%	Cip: 0%; Nor: 0%; Eno: 0%	Sut: 17.1%	Str: 24.4%; Gen: 6.1%	Fos: 0%; Col: 0%
Palmeira et al., 2016 [43]	2004–2006	S	broiler farms (18) and turkey carcasses	25 serotypes (280)	Amp: 8.0% Amc: 0.0% Cfl: 5.0% Ctf: 1.0%	Tet: 35%	Cip, Nor: 0.0% Eno: 9.0% Nal: > 60%	Sul: 3%	Gen: 12% Kn, Str: 15% Neo: 30%	Clo: 2.5% Col: 15% Fos: 5% Nit: 35% Pol: 0.0%
Bezerra et al., 2016 [59]	2014–2015	NE	broiler farms (10/1000 samples)	O:6,8 (2)	Amp: 0.0% Ctf: 100%	Tet: 100%	–	Sut: 100%	Gen: 0.0%	Clo: 100%
Borges et al., 2017 [44]	–	S	various	*S*. Enteritidis (148)	Ctf: 4.1%	Tet: 2.7%	Cip: 41.9%	Sul: 75.0% Sut: 1.4%	Gen: 6.8%	–
Koerich et al. 2018 [45]	2011–2014	S	outbreaks of fowl typhoid	*S.* Gallinarum (60)	–	Tet: 33.0%	Eno: 83.0% Nor: 90.0%	Sut: 7.0%	Neo: 30.0% Str: 62.0% Spm: 100.0%	Col: 27.0% Fos: 0.0%
Cunha-Neto et al., 2018 [54]	2014–2015	MW	slaughterhouses (1) / carcasses (850)	7 serotypes (31)	Amp, Cfl: 25.0% Atm: 21.9% Ctf: 6.3% Ctx: 18.8%	Tet: 9.4%	Cip, Eno, Nal: 0.0% Nor: 6.7%	Sul: 100% Sut: 75% Tri: 87.5%	Gen: 3.1% Str: 0.0%	Clo: 3.1% Flo, Nit: 0.0%
Baptista et al., 2018 [47]	2016	SE	slaughterhouses (6)	7 serotypes (33)	Amc: 9.1% Amp, Cef, Ctx: 12.1% Ctf: 9.1%	–	Cip, Nor: 0.0% Eno: 3.0%	–	–	–
Borges et al., 2019 [56]	–	S	–	11 serotypes (163)	Cft: 6.1%	Tet: 16%	Cip: 27%; Eno: 19%	Sox: 95.7%; Sut: 9.2%	Gen: 7.4%Spe: 12.3%	Clo: 6.1%
Penha-Filho et al., 2019 [36]	–	SE MW	chicken farms (6) and slaughterhouse (1)	36 serotypes (83)	Amc, Caz, Ctf, Ctx: 13.5% Atm: 14.5% Cfo: 6.0% Cfp: 12.0% Etp: 0.0%	Tet: 28.0%	Cip: 52.0% Eno: 31.0% Nal: 41.0%	Sut: 20.5%	–	Clo:1.2% Flo: 0.0%
*E. coli*										
Barros et al., 2012 [68]	–	NE	broiler farms (11) and laying hens farms (7) (120 samples)	*E. coli* (35)	Amo: 65.7% Cfx: 25.7%	Tet: 77.1%	Eno: 45.7% Nor: 40.0%	Sut: 65.7%	-	-
Lima-Filho et al.,2013 [63]	2013	NE	slaughterhouses (2/ 27 carcasses)	ExPEC	Amp: 81.5% Atm: 33.3% Caz: 14.8% Cfl: 88.8% Ipm: 0.0%	Tet: 100%	Cip: 44.4% Lev: 51.8%	–	Ami: 1.1% Gen: 33.3% Str: 100%	Clo: 18.5%
Gazal et al., 2015 [58]	2011–2012	S	12 farms (40 samples of avian organic fertilizers)	*E. coli* (64)	Amo: 25.3% Amp: 18.7% Atm, Ctx, Ipm: 0.0%	Tet: 35.9%	Cip, Eno, Nor: 0.0%	Sut: 12.5%	Str:17.1%	Clo, Col, Pol: 0.0%
Carvalho et al., 2015 [66]	2011–2012	S	overshoe swab samples (109 broiler houses)	*E. coli* (109)	Amp: ~55.0%	Tet: ~75%	Cip: ~35.0% Eno: ~50.0% Nal: ~80.0% Nor: ~45.0%	Sul: ~70.0% Sut: ~50.0%	Gen: ~30.0% Neo: ~25.0%	Clo: ~20.0% Flo: ~5.0% Nit: ~30.0%
Bezerra et al., 2016 [59]	2014–2015	NE	10 chicken farms (1000 samples)	*E. coli* (959)	Amp: 87.3% Ctf: 42.5%	Tet: 95.4%	Cip: 91.4%	Sut: 100%	Gen: 27.5%	Clo: 51.1% Fos: 33.3% Pol: 1.1%
Braga et al., 2016 [65]	2011–2012	SE	eight flocks from seven farms (osteomyelitis or arthritis)	APEC (15)	Amo: 73.3% Amc: 12.0% Cfl: 53.0% Cfo: 8.0% Ctf: 40.0%	Tet: 33.0%	Eno: 40.0% Nal: 68.0%	Sut: 33.0%	Gen: 20.0% Neo: 8.0%	Clo: 6.7% Pol: 0.0%
Stella et al., 2016 [61]	–	–	cloacal swabs from broilers (80) of 1 flock	APEC (15)	Amo, Amp, Cfl: 100%	Tet: 13.3%	Eno: 6.7%	Sut: 86.7%	Gen: 6.7% Neo, Str: 100%	Nit: 0.0%
	–	–		non-APEC (76)	Amo: 80.3% Amp: 81.6% Cfl: 73.7	Tet: 77.6%	Eno: 27.6%	Sut: 64.5%	Gen: 6.7% Neo: 42.1% Str: 88.2%	Nit: 5.3%
Maciel et al., 2017 [60]	–	S	avian colisepticemia outbreak (spleen and liver)	APEC (2)	Amp: 100%	Tet: 100%	Eno, Nor: 100%	Sut: 100%	Gen, Neo: 100%	–
Vaz et al., 2017 [67]	–	NE	liver of poultry carcasses (110)	*E. coli* (88)	Amc: 15.9% Atm: 19.1% Caz: 21.3% Cfl: 8.5% Ipm: 12.8%	Tet: 44.7%	Cip: 21.3%	–	Ami: 29.8% Gen:21.3% Str: 84%	
Borzi et al., 2018 [62]	–	SE	free range helmeted guineafowl (4 farms/56 cloaca, 56 oropharynges)	APEC (21)	Amc: 14.3% Amp: 71.4% Cfl: 100% Cfo: 9,5% Cro:14.3% Ctf: 4.8%	Tet: 61.9%	Cip: 23.8% Nor: 0.0%	Sut: 33.3%	Gen: 14.3% Kn: 33.3% Str: 90.5%	Clo: 9.5% Nit: 57.1%
*Campylobacter* sp.										
Ku et al., 2011 [72]	–	–	Brazilian chicken meat imported by Korea	*Campylobacter* spp. (27)	Amp: 92.6%	Tet: 51.9%	Cip, Nal: 66.7%	–	Gen:18.5%	Azi, Ery: 29.6% Cli: 25.9% Flo:7.4%
Moura et al., 2013 [76]	–	MW	poultry carcasses (92)	*Campylobacter spp. (16)*	Amo: 87.5%	Tet: 93.8%	Cip: 100% Nal: 93.8%	–	Gen, Str: 93.8%	Clo: 37.5% Ery: 68.8%
Ferro et al., 2015 [64]	–	S		*Campylobacter spp. (24)*	Amc, Ctx, Mer: 0.0% Amp: 16.7% Cfl: 98.0%;	Tet: 75.0%	Cip, Nal: 75.0%	–	Gen, Tob: 0.0%	Clo: 4.16% Ery: 0.0%
Melo et al., 2019 [73]	2011–2012; 2015–2016	SE	poultry carcasses (1070)	*C. jejuni* (2011-2012/55) (2015-2016/44)	2011–2012 Amc: 65.5% 2015-2016 Amc: 43.2%	2011–2012 Tet: 74.5% 2015-2016 Tet:81.8%	-	-	2011–2012 Gen: 14.5% 2015–2016 Gen: 2.3%	2011–2012 Ery: 38.2% 2015–2016 Ery: 9.1%

^a^ Geographic Region: S (South), SE (Southeast), MW (Midwest), N (North), NE (Northeast); ^b^ Ami: amikacin; Amc: amoxicillin + clavulanic acid; Amo: amoxicillin; Amp: ampicillin; Atm: aztreonam; Azi: azithromycin; Caz: ceftazidime; Cip: ciprofloxacin; Cfo: cefoxitin; Cfl: cephalothin; Cfp: cefepime; Cro: ceftriaxone; Clo: chloramphenicol; Cli: clindamycin; Col: colistin; Ctf: ceftiofur; Ctx: cefotaxime; Dox: doxycycline; Eno: enrofloxacin; Ery: erythromycin; Etp: ertapenem; Flo: florfenicol; Gen: gentamicin; Ipm: imipenem; Kn: kanamycin; Lev: levofloxacin; Mer: meropenem; Nal: nalidixic acid; Neo: neomycin; Nor: norfloxacin; Nit: nitrofurantoin; Pol: polymyxin B; Spe: spectinomycin; Spm: spiramycin; Str: streptomycin; Sul: sulfonamide; Sut: sulfamethoxazole-trimethoprim; Tet: tetracycline; Tob: tobramycin; Tri: trimethoprim.

**Table 2:** Resistance profile of *Salmonella* sp. *Escherichia coli* and *Yersinia enterocolitica* isolated from pigs, Brazil (data published between 2009 and 2019).

Ref.	Sampling Period	Geographic Region ^a^	Local (n)	Isolate (n)	Antimicrobial Resistance^b^					
					Beta-lactam	Tetracycline	Quinolone	Sulfonamide	Aminoglycoside	Others
*Salmonella* sp.										
Kich et al., 2011 [81]	2007	S	various	8 serotypes (572)	Amc: 1.0% Amp: 46.6% Cfl: 5.0% Cfo: 1.0%	Tet: 79.0%	Nal: 5.0%	Sul: 23.0% Sut: 10.0%	Gen: 39.0% Kn: 41.0% Str: 35.0%	Clo: 10.0%
Morales et al., 2012 [88]	–	–	swine herds	*S. enterica* (124)	–	–	–	–	–	Col: 21.0%
Lopes et al., 2015 [80]	2008–2011	S	slaughterhouses (1)/ intestinal content and carcasses	28 serotypes (225)	Amp: 29.8%	Tet: 54.5%	Cip: 0.9% Nal: 33.3%	Sul: 39.6% Str: 33.7% Tri: 8.0%	Gen: 10.7% Kn: 14.7%	Clo: 14.2%
Almeida et al., 2016 [83]	2000–2012	S	various	*S*. Typhimurium (22)	Amp: 81.4%	Tet: 62,9%	Cip, Lev: 3.0% Nal: 59.0%	Sut: 66.6%	–	Clo: 74.0%
Souto et al., 2017 [84]	2011–2014	SE	fecal samples	*Salmonella* sp. (39)	Amo: 89.7% Amp: 82.0% Cfo: 2.6 %	Tet: 97.4%	Nal: 33.3% Nor: 2.6 %	Sut: 53.8%	Gen: 87.1%	–
Rau et al., 2018 [95]	2011–2017	S	animal products (40)	*Salmonella* sp. (40)	–	–		v	–	Col: 1 isolate (*mcr-*1 positive)
Viana et al., 2019 [85]	–	–	pork production chain	25 serotypes (280)	Amp: 81.0% Caz, Cfo: 4.8%	Tet: 88.1%	Cip: 50.0%	Sut: 19.0%	Gen: 16.7%Str: 90.5%	Clo: 71.4%
*E. coli*										
Morales et al., 2012 [88]	–	–	swine herds	ETEC (126)	–	–	–	–	–	Col: 6.3%
Silva et al., 2016 [87]	2012	–	swine herds	*E. coli* (267)	Ctf: eight isolates (CTX-M-15-producing)	–	–	–	–	–
Kiefer et al., 2018 [89]	–	–	swine herd (126)	colistin-resistant *E. coli* (8)	–	–	–	–	–	Col: colistin-resistant E. coli
Spindola et al., 2018 [86]	–	SE	swine urine (300)	*E. coli* (186)	Amc: 1.1% Amp: 80.1% Cfo: 1.1% Ctf: 2.6%	Tet: 91.9%	Cip: 22.5% Eno: 33.3% Nal: 66.1% Nor: 21.5%	Sul: 94.6% Sut: 54.6%	Gen: 2.6% Spe: 11.2% Str: 52.6%	Flo: 83.3%
*Yersinia enterocolitica*										
Ruzak et al., 2014 [92]	2005–2011	SE, NE, S	various	*Y. enterocolitica* (60)	Amp: 100% Cfl: 97.0% Cfo: 13.0%	Tet: 8.0%	–	Sul: 68.0% Sut: 10.0% Tri: 12.0%	Ami: 2.0%	-
Frazão et al., 2017 [93]	1979–2012	–	various	*Y. enterocolitica* (39)	Amc: 55.8% Cfo, Cfz: 100% Amp, Tic: 94.0%	–	–	–	–	-
Martins et al., 2018 [91]	–	SE	Pig farm (2/20 samples); slaughterhouse (1/960 samples	*Y. enterocolitica * (16)	Amo, Amp, Ipm: 100%	Tet: 12.5%	Nal: 100.0%	Sul: 100.0%	Gen: 37.5 Neo: 100% Str: 100%	

^a^ Geographic Region: S (South), SE (Southeast) and MW (Midwest); ^b^ Ami: amikacin; Amc: amoxicillin + clavulanic acid; Amo: amoxicillin; Amp: ampicillin; Caz: ceftazidime; Cip: ciprofloxacin; Cfo: cefoxitin; Cfl: cephalothin; Cfz: ceftazidime; Clo: chloramphenicol; Col: colistin; Ctf: ceftiofur; Eno: enrofloxacin; florfenicol; Gen: gentamicin; Ipm: imipenem; Kn: kanamycin; Lev: levofloxacin; Nal: nalidixic acid; Neo: neomycin; Nor: norfloxacin; Nit: nitrofurantoin; Rif: rifampicin; Spe: spectinomycin; Str: streptomycin; Sul: sulfonamide; Sut: sulfamethoxazole-trimethoprim; Tet: tetracycline; Tic: ticarcillin; Tri: trimethoprim.

**Table 3:** Resistance profile of *Staphylococcus* spp, *Salmonella* sp., *Escherichia coli,* and *Listeria monocytogenes* isolated from dairy and beef cattle, Brazil (data published between 2009 and 2019).

Ref.	Sampling period	Region ^a^	Local (n)	Isolate ^b^(n)	Antimicrobial Resistance ^c^					
					Beta-lactam	Tetracycline	Quinolone	Sulfonamide	Aminoglycoside	Others
*Staphylococcus* sp.										
Ceotto et al., 2009 [106]	–	SE	dairy herd	*S. aureus* (46) **	Amp: 67.4% Oxa: 0.0% Pen G: 65,2%	Tet: 41.3%	Cip:10.9%	–	Gen:15.2%	Cli: 13.1% Ery: 58.7%
Laport et al., 2012 [107]	1995–2003	SE	dairy herd (21)	CNS (49) ***	Oxa: 6.1% Pen: 51.0%	Tet: 14.3%	Cip: 2,0%	Sut: 10.2%	Gen: 2,0%	Cli: 12.2% Ery: 18.4% Rif: 0.0%
Costa et al., 2012 [114]	–	–	dairy herd (38)	*S. aureus* (352) **	Amp: 81.4% Oxa: 2.0% Pen: 82.3%	Tet: 16.7%	Eno: 0.3%	Sut: 6.3%	Gen: 1.7% Neo: 3.4%	Clo: 1.7% Flo: 0.3% Lin: 7.9% Nit: 0.0% Nov: 1.4%
Silva et al., 2013 [117]	–	SE	dairy herd (11)	*S. aureus* (56)***	Cfl, Oxa: 0.0%	Tet: 3.5%	Cip: 0.0%	Sut: 0.0%	Gen, Tob: 0.0%	Cli, Ery: 0.0% Clo: 3,5%
										
Silva et al., 2014 [132]	–	SE	dairy herd	CNS (128) ***	Cfl, Oxa: 20.3%	–	–	–	–	–
da Costa Krewer et al., 2015 [100]	–	NE	dairy herd (8)	*S. aureus* (126) ** oCPS (61) CNS (31)	Amp: 67.0% Amo: 67.4% Oxa: 1.8% Pen: 66.0%	Dox: 11.4% Tet: 17.4%	Cip: 0.9% Eno: 0.5%	Sut: 2.2%	Gen: 0.5% Str: 11.9%	Ery, Lin: 1.8% Rif: 0.0%
Castelani et al., 2014 [111]	2009-2010	SE	dairy herd (2)	S. aureus * (110: 83 from heifers and 27 from cows)	Heifers Amp: 14.5% Oxa: 0.0% Pen: 39.6% Cows Amp: 40.7% Oxa: 0.0% Pen: 62.9%	–	–	v	Heifers Gen, Kn: 0% Neo: 8.4% Cows Gen, Kn: 0% Neo: 7.4%	Flo: 0.0%
Fernandes dos Santos et al., 2016 [109]	2008–2010	NE, S, SE	dairy herd (48)	*S. aureus* (79) * 91 CNS (91)	*S. aureus* Oxa: 0.0% Pen: 30.4% CoNS Oxa: 47.0% Pen: 34.1%;	*S. aureus * Tet: 8.9% CNS Tet: 24.2%	*S. aureus * Eno MIC_90_ 0.06-0.5 CNS Eno MIC_90_ 0.06-32	*S. aureus * Sul: 1.3% Sut: 0.0% CNS Sul: 4.4% Sut: 2.2%	*S. aureus * Gen: 0% CNS Gen: 6.6%	*S. aureus * Ery: 1.3% Cli MIC_90_ 0.125 CNS Ery: 13.2% Cli MIC_90_ 0.25
Marques et al., 2017 [110]	2012	SE	dairy herd (3)	*S. aureus* (20) ***	Amo: 5.0% Amp: 25.0% Oxa: 0.0% Pen: 100%	Tet: 5.0%	Cip: 25.0% Eno, Moxi: 20.0%	Sut: 35.0%	Neo: 15.0% Str: 25.0%	Azi, Clo: 20.0% Ery: 10.0% Nov: 30.0%
Mello et al., 2017 [118]	–	6 states	dairy herd	*S. aureus* (82) *** others (99)	Oxa: 18.2% (1 *S. aureus*) *S. aureus * MIC_50_ 0.094 MIC_90_ 0.25 Others MIC_50_ 0.25 MIC_90_ 1.50					Van: 0.0% *S. aureus * MIC_50_ 0.5 MIC_90_ 1.0 Others MIC_50_ 1.0 MIC_90_ 1.5 hR: 7.1% (1 *S. aureus*)
Guimarães et al., 2017 [119]	–	SE	dairy herd (1)	*S. aureus* (60) **	MRSA: 23.3% OS-MRSA: 25.0% MSSA: 51.7%					
Haubert et al., 2017 [112]	–	S	dairy herd	*S. aureus* (31) **	Amp: 52.0% Cef: 19.0% Oxa: 42.0% Pen: 48.0%	Tet: 39.0%	Eno: 6.0%	Sul: 65.0%	Str: 16.1% Tob: 29.0%	Cli: 52.0% Ery: 35.0% Tri: 0.0%
Martini et al., 2017 [108]	–	SE	dairy herd (10)	*S. aureus* (266) *	Amp: 66.5% Oxa: 0.0% Pen: 70.7%	Tet: 27.4%				
Freitas et al., 2018 [113]	–	S	dairy herd	*S. aureus* (27) *** CNS (3)	Amo: 50.0% Amp: 43.3% Pen: 70.0%	Tet: 96.7%	Eno: 43.3% Nor: 6.7%		Gen: 86.7% Neo: 96.7%	B: 43.3% Tri: 100%
*E. coli*										
Fernandes et al., 2017 [127]	2014	-	industry (beef jerky) (1)/ processing surfaces	2	Amc, Ctx, Ipm: 0% Amp, Cef: 50%	Tet: 50%	Cip: 50% (I)	Sut: 50%	Ami, Gen: 0% Str: 50% (I)	Clo, Nal: 50.0% Tri: 0.0%
Santos et al., 2018 [126]	2015	SE	slaughterhouse (1)/carcasses	18 STEC	Amp, Cef, Caz, Imp: 0%	Tet: 0%	Cip 0%	Sut 0%	Gen, Str: 0%	Clo, Nal, Nit: 0.0%
*Salmonella* spp.										
Cossi et al., 2013 [129]	v	MW	butcher shops (3)/environment, equipment and employee hands	7 (cutting board surfaces)	Ctx: 0% Cfo: 29% Cef: 29%, 14% (I) Ipm: 14%	Min: 71%, 14% (I) Tet: 86%	–	Sul, Sut: 86%	Ami: 0% Kn: 14% Tob: 29%, 14% (I)	–
da Silva et al., 2014 [130]	2009–2010	S	Slaughterhouse (1)/carcasses (120)	6	Amp, Cef, Cfo, Ctx, Ipm: 0%	Tet: 0%	Cip: 0%	Sul, Sut: 0%	Ami, Gen, Kn, Str: 0%	Clo, Nal: 0%
Loiko et al., 2016 [128]	2010–2012	S	Slaughterhouse (1)/carcasses (108)	1	Amp, Cef, Cfo: 100% Ctx, Ipm: 0%	Tet: 0%	Cip: 0%	Sul, Sut: 0%	Ami, Gen, Kn, Str: 0%	Clo: 0% Nal: 100% (I)
Fernandes et al., 2017 [127]	2014	-	industry (beef jerky) (1)/environment and food	1 (processing surfaces) 3 (raw material)	Amp, Amc, Cfo, Cef, Ctx, Ipm: 0%	Tet: 0%	Cip: 0%	Sut: 25%	Ami, Gen, Str: 0%	Clo, Nal, Tri: 0%
*Listeria monocytogenes*										
Camargo et al., 2014 [133]	–	SE	slaughterhouse (2)/animals and carcasses (209)	5	Amp: 0%	Tet: 0%	–	–	Gen: 0%	Ery, V: 0%
Camargo et al., 2015 [131]	1978–2013	11 states	–	69 (from carcass and food-processing environments), 43 (from beef food) and 25 (from clinical cases)	Imp, Pen: 0% Oxa: 57%, 17% (I)	Tet: 0%	–	Sut: 0%	Gen: 0%	Clo, Ery, Rif, V: 0% Cli: 53%, 36% (I)
Loiko et al., 2016 [128]	2010–2012	S	slaughterhouse (1)/carcasses (108)	7	Amp, Ipm: 0% Cef: 82%, Cfo: 91% Ctx: 100% Ipm: 0%	Tet, Min: 0%	Cip: 0%	Sul: 55% Sut: 0%	Ami, Gen, Kn: 20–10% Tob: ~30%	Clo, Ery, Tri, V: 0% Nal: 100%

^a^ Geographic region: S (South), SE (Southeast), NE (Northeast) and MW (Midwest); ^b^ CNS: coagulase-negative *Staphylococcus;* oCPS: other coagulase-positive *Staphylococcus;* STEC: shiga toxin-producing *E. coli*; between parenthesis: number of isolates tested; ^c^ Ami: amikacin; Amc: amoxicillin + clavulanic acid; Amo: amoxicillin; Amp: ampicillin; Azi: azithromycin; B: bacteriocin; Caz: ceftazidime; Cip: ciprofloxacin; Cfo: cefoxitin; Cfl: cephalothin; Clo: chloramphenicol; Cli: clindamycin; Ctx: cefotaxime; Dox: doxycycline; Eno: enrofloxacin; Ery: erythromycin; Flo: florfenicol; Gen: gentamicin; Ipm: imipenem; Kn: kanamycin; Lin: lincomycin; Min: minocycline; Moxi: moxifloxacin; Nal: nalidixic acid; Neo: neomycin; Nov: novobiocin; Nor: norfloxacin; Nit: nitrofurantoin; Oxa: oxacillin; Pen: penicillin G; Rif: Rifampicin; Str: streptomycin; Sul: sulfonamide; Sut: sulfamethoxazole-trimethoprim; Tet: tetracycline; Tob: tobramycin; Tri: trimethoprim, Van: vancomycin; MIC50: minimum concentration required to inhibit 50% of bacterial isolates (µg/mL); MIC90: minimum concentration required to inhibit 90% of bacterial isolates (µg/mL); MRSA: Methicillin-resistant *S. aureus*; MSSA: Methicillin-sensitive *S. aureus*; * clinical form not reported, ** from clinical and sub-clinical mastitis, *** from subclinical mastitis.

**Table 4:** Antimicrobial resistance genes detected among *Staphylococcus* spp. isolates recovered from bovine mastitis, Brazil (data published between 2009 and 2019).

Reference	Bacterial Species ^a^	Year of Samples Isolation	Region ^b^	Antimicrobial Resistance Gene				
				Beta-lactam	Tetracycline	MLSB^c^	Aminoglycoside	Others
Laport et al., 2012 [107]	*S. chromogenes, S. sciuri, S. xylosus*	–	SE	*mec*A	–	–	–	–
Silva et al., 2013 [117]	*S. aureus*	–	SE	–	*tet*(K)	–	–	*fex*A
Silva et al., 2014b [132]	CNS, oCNP	–	–	*bla*Z, *mec*A (*S. epidermidis, S. chromogenes, S. warneri, S. hyicus, S. simulans)*	*tet*(K) (*S. epidermidis, S. chromogenes, S. warneri)*	*erm*C (*S. epidermidis*); *lnu*B, *lsa*E (*S. chromogenes*)	*ant(4’)-Ia* (*S. epidermidis, S. chromogenes, S. warneri*); *aac(6’)-aph(2”)* (*S. epidermidis, S. warneri); aadE* (*S. chromogenes*); *str* (*S. hyicus, S. warneri, S. epidermidis)*	*gyr*A, *grl*A (mutation)
da Costa Krewer et al., 2015 [100]	*S. aureus*	2004–2008	NE	*bla*Z, *mec*A	–	–	–	–
Fernandes dos Santos et al., 2016 [109]	*S. epidermidis*	2008–2010	SE, S, NE	*mec*A	–	–	–	-
Martini et al., 2017 [108]	*S. aureus*	–	SE	*bla*Z	*tet*(K), *tet*(L), *tet*(M), *tet*(O)	–	–	–
Guimarães et al., 2017 [119]	*S. aureus*	–	SE	*mec*A	–	–	–	–
Haubert et al., 2017 [112]	*S. aureus*	–	S	*bla*Z	*tet*(B), *tet*(K), (*tet*)L, (*tet*)M	*erm*B, *erm*C, *ere*B	*str*A, *str*B	*dfr*A, *dfr*G
Marques et al., 2017 [110]	*S. aureus*	–	SE	*bla*Z, *mec*A	–	–	–	–
Mello et al., 2017 [118]	*S. aureus, S. chromogenes, S. S. epidermidis, S. haemolyticus, S. saprophyticus, S. simulans, S. xylosus, S. warneri*	–	6 states	*mec*A	–	–	–	–

^a^ CNS: coagulase-negative *Staphylococcus;* oCPS: other coagulase-positive *Staphylococcus;* ^b^ Geographic region: S (South), SE (Southeast) and NE (Northeast); ^c^ MLSB: macrolides, lincosamides and streptogramin B.

**Table 5:** Occurrence of ESBL and pAmpC-encoding genes in isolates from animals or animal-derived foods, Brazil (2009–2019).

Ref.	Year of Isolation	Source	Country/ Region^a^	Bacterial Species	β-Lactamase Genes or Group of Genes Found (*bla*)
Mattiello et al., 2015 [181]	2002–2012	Poultry producing environment and by-product meals	Brazil/SE	*Salmonella* Schwarzengrund, *Salmonella enterica*, *Salmonella* Infantis, *Salmonella* Senftenberg, *Salmonella* Montevideo, *Salmonella* Cerro, *Salmonella* Worthington, *Salmonella* Heidelberg	*TEM, CTX-M, CMY*
Fernandes et al., 2009 [155]	2004	Poultry	Brazil/SE	*Salmonella* Typhimurium	*CTX-M-2*
Fitch et al., 2016 [141]	2004–2011	Poultry during slaughter	Brazil/MW, S	*Salmonella* Agona, *Salmonella* Brackenrindge, *Salmonella* Emek, *Salmonella* Enteritidis, *Salmonella* Gaminara, *Salmonella* Give, Salmonella GroupIII, Salmonella Hadar, *Salmonella* Heidelberg, *S*. Infantis, *Samonella* Minnesota, *Salmonella* Newport, *Salmonella* Panama, *Salmonella* Poona, *Salmonella* Rissen, *Salmonella* Saintpaul, *Salmonella* Schwarzengrund*/,* Bredeney, and *S.* Typhimurium, *Salmonella* Weslaco	*TEM, CTX-M-1, CTX-M-2, CTX-M-8, CTX-M-14, CMY-2*
Moura et al., 2018 [138]	2008–2015	Chicken and turkey meat; swine feces	Brazil/MW, SE, S	*S.* Agona, *S.* Typhimurium, S. Minnesota, *S.* Heidelberg, S. Infantis	*CTX-M-2, CTX-M-8, CMY-2*
Penha Filho et al., 2019 [36]	2009–2012	Poultry at farms	Brazil/MW, SE	*S.* Schwarzengrund, *S.* Newport*, S.* Heidelberg	*CTX-M-2, CMY-2*
Moura et al., 2017 [156]	2010	Chicken meat	Brazil/MW	*S. Minnesota*	*CMY-2*
Botelho et al., 2015 [140]	2010–2011	Chicken carcasses (frozen)	Brazil/SE	*Escherichia. coli*	*CTX-M-1, CTX-M-2, CTX-M-8, CMY-2*
Ferreira et al., 2014, 2016, 2017; Galetti, 2019 [137,139,143,178]	2011–2012	Poultry cloacal swabs	Brazil/SE	*E. coli, E. fergusonii, K. pneumoniae*	*CTX-M-2, CTX-M-8, CTX-M-15, CMY-2*
Casella et al., 2015 [136]	2011, 2013	Chicken meat	Brazil/SE	*Proteus mirabilis, Citrobacter diversus, Klebsiella pneumoniae, and E. coli*	*TEM, SHV, CTX-M-2, CTX-M-8*
Fernandes et al., 2017 [127]	2012	Poultry meat	Brazil/SE	*Salmonella* Muenchen*, S.*Typhimurium*, Salmonella* Corvallis	*CTX-M-2, CTX-M-8*
Ibbe et al., 2017 [142]	2012–2013	Chicken (live and carcasses)	Brazil/MW, S	*S.* Minnesota*, S.* Hilderberg	*CTX-M-8, ACC-1, CMY-2*
Koga et al., 2015a,b [69,148]	2013	Chicken carcasses (refrigerated)	Brazil/S	*E. coli*	*SHV, CTX-M-1, CTX-M-2, CTX-M-8, CIT*
Cyoia et al., 2019 [149]	2013–2014	Chicken carcasses	Brazil/S	*E. coli*	*TEM, SHV, CTX-M-1, CTX-M-2, CTX-M-8*
Cunha et al., 2017 [147]	2013–2016	Poultry cloacal swabs	Brazil/S	*E. coli*	*TEM, CTX-M-2, CTX-M-55, CMY-2*
Casella et al., 2018 [146]	2014	Chicken meat and cloacal swabs	Brazil/SE	*E. coli*	*CTX-M-2, CTX-M-8, CTX-M-15, CTX-M-55, CMY-2*
Hoepers et al., 2018 [153]	2014–2015	Turkeys with clinical signs	Brazil/ MW, SE, S	*E. coli*	*TEM, CTX-M-2, CTX-M-8, CMY-2*
Tiba Casas et al., 2019 [144]	2014–2016	Poultry and poultry meat	Brazil/SE	*S.* Heidelberg	*CMY-2*
Zogg et al., 2016, [150]	2015	Chicken carcasses (frozen)	Swiss	*E. coli*	*CTX-M-2, CTX-M-8*
Nahar et al., 2018 [151]	2015	Chicken meat	Japan	*E. coli*	*TEM, CTX-M-1, CTX-M-2, CTX-M-8*
Brisola et al., 2019 [179]	2016-2017	Swine feces	Brazil/SC	*E. coli*	*TEM, CMY-2*
Kim et al., 2018 [152]	n.d.	Chicken meat	South Korea	*E. coli*	*TEM, CTX-M-2, CTX-M-94, OXA-1*

^a^ Geographic region: S (South), SE (Southeast) and MW (Midwest)

## Appendix A

**Table A1:** Specific current legislation in Brazil for the use of antimicrobials in animal production.

Legislation	Public Agency *	Year	Objective	Reference
Circular Letter nº 047/1998	MAPA	1998	Prohibits the use of avoparcin for growth promoter or animal performance enhancer purposes.	[11]
Normative Instruction N.º 42	MAPA	1999	Change the National Plan for the Control of Residues in Products of Animal Origin - PNCR and the Programs for the Control of Residues in Meat - PCRC, Honey - PCRM, Milk - PCRL and Fish - PCRP.	[12]
Ordinance Nº 31	MAPA	2002	Prohibits the use of arsenicals and antimonial active ingredients in the manufacture of products intended for animal feed, for growth promoters or animal performance improvers.	[13]
Normative Instruction N.º 09	MAPA	2003	Prohibits the use of chloramphenicol and nitrofurans, and products containing these active ingredients for veterinary use, and susceptible to feeding to all animals and insects.	[14]
Normative Instruction N.º 11	MAPA	2004	Prohibits the manufacture, import, sale, and use of the chemical called olaquindox, as a growth-promoting additive in food-producing animals.	[15]
Normative Instruction N.º 35	MAPA	2005	Prohibits the use of feed products containing the chemical called carbadox.	[16]
Normative Instruction Nº 26	MAPA	2009	Approves the technical regulation for the manufacture, quality control, marketing and employment of veterinary antimicrobial products, and determines that amphenicols, tetracyclines, beta-lactams (systemic benzyl penicillamines and cephalosporins), quinolones, and systemic sulfonamides are for use exclusively in veterinary antimicrobial products, and are prohibited for use as performance-enhancing zootechnical additives, or as food preservatives.	[17]
Ordinance N.º 396	MAPA	2009	Establishes responsibilities of the units of the Secretariat of Agricultural Defense (SDA) involved in the PNCRC/MAPA research subprogram.	[183]
Normative Instruction Nº 14	MAPA	2012	Prohibits the import, manufacture, and use of antimicrobial substances spiramycin and erythromycin throughout the national territory for zootechnical additive to improve performance in animal feed.	[18]
ANVISA—Resolution Nº 53 - Internalize the Resolution Mercosul N.º 54/2000	MS	2012	Approves the maximum residue levels of veterinary medicines in animal food.	[184]
Codex Alimentarius—N.º 02/2015	FAO-OMS/ANVISA	2015	Updates maximum residue limits for veterinary food products.	[185]
Normative Instruction Nº 45	MAPA	2016	Prohibits, throughout the national territory, the import and the manufacture of the antimicrobial substance colistin sulfate, with the purpose of a performance-enhancing feed animal additive.	[19]
Normative Instruction Nº 54	MAPA	2018	Approves the technical regulation for the registration of performance-enhancing antimicrobial additives and anticoccidial feed additives.	[186]
ANVISANormative Instruction Nº 51	MS	2019	Establishes the list of maximum residue limits (LMR), acceptable daily intake (IDA) and acute reference dose (DRfA) for active pharmaceutical ingredients (IFA) of veterinary drugs in foods of animal origin.	[187]
Normative Instruction N^o^ 1	MAPA	2020	Prohibits, throughout the national territory, the importation, manufacture, sale, and use of performance-enhancing additives containing the antimicrobial agents tylosin, lincomycin, and tiamulin, classified as important in human medicine.	[20]

* MAPA, Ministry of Agriculture and Livestock; MS, Ministry of Health; FAO, Food and Drug Organization; OMS, World Health Organization; ANVISA, National Sanitary Vigilance Agency

## References

[1] Alexandratos N., Bruinsma J. (2012). World Agriculture Towards 2030/2050: The 2012 Revision.

[2] Tomley F.M., Shirley M.W. (2009). Livestock infectious diseases and zoonoses. Philos. Trans. R. Soc. B Biol. Sci..

[3] Tang K.L., Caffrey N.P., Nóbrega D.B., Cork S.C., Ronksley P.E., Barkema H.W., Polachek A.J., Ganshorn H., Sharma N., Kellner J.D. (2017). Restricting the use of antibiotics in food-producing animals and its associations with antibiotic resistance in food-producing animals and human beings: A systematic review and meta-analysis. Lancet Planet. Health.

[4] Tang K.L., Caffrey N.P., Nóbrega D.B., Cork S.C., Ronksley P.E., Barkema H.W., Polachek A.J., Ganshorn H., Sharma N., Kellner J.D. (2019). Comparison of different approaches to antibiotic restriction in food-producing animals: Stratified results from a systematic review and meta-analysis. BMJ Glob. Health.

[5] World Organization for Animal Health (OIE) (2018). Annual Report on Antimicrobial Agents Intended for Use in Animals: Better Understand the Global Situation.

[6] Word Health Organization (WHO) Global Action Plan on Antimicrobial Resistance. https://www.who.int/antimicrobial-resistance/publications/global-action-plan/en/.

[7] World Organization for Animal Health (OIE) (2016). The OIE Strategy on Antimicrobial Resistance and the Prudent Use of Antimicrobials. https://www.oie.int/fileadmin/Home/eng/Media_Center/docs/pdf/PortailAMR/EN_OIE-AMRstrategy.pdf.

[8] Castanon J.I.R. (2007). History of the use of antibiotic as growth promoters in European poultry feeds. Poult. Sci..

[9] Food and Drug Administration (FDA) Timeline of FDA Action on Antimicrobial Resistance. https://www.fda.gov/animal-veterinary/antimicrobial-resistance/timeline-fda-action-antimicrobial-resistance.

[10] Antimicrobial Resistance and Animals—Actions. https://www.canada.ca/en/public-health/services/antibiotic-antimicrobial-resistance/animals/actions.html.

[11] Brazil Ministério da Agricultura, Pecuária e Abastecimento (MAPA) Substâncias Proibidas na Alimentação Animal Com a Finalidade de Aditivo e Legislação Correspondente. http://www.agricultura.gov.br/assuntos/insumos-agropecuarios/insumos-pecuarios/arquivos-de-insumos-pecuarios/PortalMAPASubstanciasproibidasmaio2012.pdf/view.

[12] Brazil Ministério da Agricultura, Pecuária e Abastecimento (MAPA) Instrução Normativa SDA No 42, de 20 de Dezembro de 1999. https://www.agricultura.gov.br/assuntos/inspecao/produtos-animal/plano-de-nacional-de-controle-de-residuos-e-contaminantes/documentos-da-pncrc/instrucao-normativa-sda-n-o-42-de-20-de-dezembro-de-1999.pdf/view.

[13] Brazil Ministério da Agricultura, Pecuária e Abastecimento (MAPA) Portaria no 31, de 29 de Janeiro de 2002. https://www.agricultura.gov.br/assuntos/insumos-agropecuarios/insumos-pecuarios/alimentacao-animal/arquivos-alimentacao-animal/legislacao/portaria-no-31-de-29-de-janeiro-de-2002.pdf/view.

[14] Brazil Ministério da Agricultura, Pecuária e Abastecimento (MAPA) Instrução Normativa SDA No 09, de 27 de Junho de 2003. https://www.agricultura.gov.br/assuntos/inspecao/produtos-animal/plano-de-nacional-de-controle-de-residuos-e-contaminantes/documentos-da-pncrc/instrucao-normativa-sda-n-o-09-de-27-de-junho-de-2003.pdf/view.

[15] Brazil Ministério da Agricultura, Pecuária e Abastecimento (MAPA) Instrução Normativa SDA No 11, de 24 de Novembro de 2004. https://www.agricultura.gov.br/assuntos/inspecao/produtos-animal/plano-de-nacional-de-controle-de-residuos-e-contaminantes/documentos-da-pncrc/instrucao-normativa-sda-n-o-11-de-24-de-novembro-de-2004.pdf/view.

[16] Brazil Ministério da Agricultura, Pecuária e Abastecimento (MAPA) Instrução Normativa SDA No 35, de 14 de Novembro de 2005. https://www.agricultura.gov.br/assuntos/inspecao/produtos-animal/plano-de-nacional-de-controle-de-residuos-e-contaminantes/documentos-da-pncrc/instrucao-normativa-sda-n-o-35-de-14-de-novembro-de-2005.pdf/view.

[17] Brazil Ministério da Agricultura, Pecuária e Abastecimento (MAPA) Instrução Normativa No 26, de 9 de Julho de 2009. https://www.agricultura.gov.br/assuntos/insumos-agropecuarios/insumos-pecuarios/alimentacao-animal/arquivos-alimentacao-animal/legislacao/instrucao-normativa-no-26-de-9-de-julho-de-2009.pdf/view.

[18] Brazil Ministério da Agricultura, Pecuária e Abastecimento (MAPA) Instrução Normativa No 14, de 17 de Maio de 2012. https://www.agricultura.gov.br/assuntos/insumos-agropecuarios/insumos-pecuarios/alimentacao-animal/arquivos-alimentacao-animal/legislacao/instrucao-normativa-no-14-de-17-de-maio-de-2012.pdf/view.

[19] Brazil Ministério da Agricultura, Pecuária e Abastecimento (MAPA) Instrução Normativa No 45, de 22 de Novembro de 2016. https://www.agricultura.gov.br/assuntos/insumos-agropecuarios/insumos-pecuarios/alimentacao-animal/arquivos-alimentacao-animal/legislacao/instrucao-normativa-no-45-de-22-de-novembro-de-2016.pdf/view.

[20] Brazil Ministério da Agricultura, Pecuária e Abastecimento (MAPA) Instrução Normativa No 01, de 13 de Janeiro de 2020. https://www.in.gov.br/en/web/dou/-/instrucao-normativa-n-1-de-13-de-janeiro-de-2020-239402385.

[21] World Health Organization (WHO) Critically Important Antimicrobials for Human Medicine, 5th Revision. https://www.who.int/foodsafety/publications/antimicrobials-fifth/en/.

[22] Brazil Agência Nacional de Vigilância Sanitária (ANVISA) (2018). Limites Máximos de Resíduos de Medicamentos Veterinários em Alimentos de Origem Animal: Documento de Base para Discussão Regulatória.

[23] Brazil Agência Nacional de Vigilância Sanitária (ANVISA) Programa de Análise de Resíduos de Medicamentos Veterinários em Alimentos de Origem Animal. https://portal.anvisa.gov.br/documents/33916/395364/PAMVet-+Monitoramento+de+Res%C3%ADduos+em+Leite+Exposto+ao+Consumo+-+Relatório+2006-2007/4777c371-e5b5-42e0-9c3f-43670009a802.

[24] Brazil Ministério da Agricultura, Pecuária e Abastecimento (MAPA) Instrução Normativa No 5, de 23 de Abril de 2019. https://www.agricultura.gov.br/assuntos/inspecao/produtos-animal/plano-de-nacional-de-controle-de-residuos-e-contaminantes/InstruoNormativaN05.2019PNCRC2019.pdf/view.

[25] Brazil Agência Nacional de Vigilância Sanitária (ANVISA) Relatório do Monitoramento da Prevalência e do Perfil de Suscetibilidade Aos Antimicrobianos em Enterococos e Salmonelas Isolados de Carcaças de Frango Congeladas Comercializadas No Brasil. http://portal.anvisa.gov.br/documents/33916/395481/Relatório+Prebaf+-+Programa+Nacional+de+Monitoramento+da+Prevalência+e+da+Resistência+Bacteriana+em+Frango/f6bb5296-e633-4f7b-b81f-48a99430da6a?version=1.1&download=true.

[26] Food and Agriculture Organization (FAO) (2018). Monitoring Global Progress on Addressing Antimicrobial Resistance: Analysis Report of the Second Round of Results of AMR Country Self-Assessment Survey 2018.

[27] Wall B.A., Mateus A., Marshall L., Pfeiffer D., Lubroth J., Ormel H.J., Otto P., Patriarchi A., Food and Agriculture Organization of the United Nations (FAO) (2016). Drivers, Dynamics and Epidemiology of Antimicrobial Resistance in Animal Production.

[28] Associação Brasileira de Proteína animal (ABPA) (2018). Relatório Anual. https://abpa-br.com.br/storage/files/relatorio-anual-2018.pdf.

[29] Minafra e Rezende C.S., de Mesquita A.J., Andrade A.M., Linhares G.F.C., de Mesquita A.Q., Minafra C.S. (2005). Serovars of Salmonella isolated from carcasses of broilers slaughtered in the State of Goiás, Brazil, and their resistance profiles to antibioctics. Rev. Port. Cien. Vet..

[30] Dias de Oliveira S., Siqueira Flores F., dos Santos L.R., Brandelli A. (2005). Antimicrobial resistance in *Salmonella enteritidis* strains isolated from broiler carcasses, food, human and poultry-related samples. Int. J. Food Microbiol..

[31] Cardoso M.O., Ribeiro A.R., dos Santos L.R., Pilotto F., de Moraes H.L.S., Salle C.T.P., da Silveira Rocha S.L., Nascimento V.P. (2006). Do antibiotic resistance in *Salmonella enteritidis* isolated from broiler carcasses. Braz. J. Microbiol..

[32] Ribeiro A.R., Kellermann A. (2006). Resistência antimicrobiana em *Salmonella enterica* subsp*. enterica sorovar Hadar* isoladas de carcaças de frango. Arq. Inst. Biol..

[33] Ribeiro A.R., Kellermann A., Santos L.R., Nascimento V.P. (2008). Resistência antimicrobiana em *Salmonella enteritidis* isoladas de amostras clínicas e ambientais de frangos de corte e matrizes pesadas. Arq. Bras. Med. Vet. Zootech..

[34] Oliveira W.F., Cardoso W.M., Salles R.P.R., Romão J.M., Teixeira R.S.C., Câmara S.R., Siqueira A.A., Marques L.C.L. (2006). Initial identification and sensitivity to antimicrobial agents of *Salmonella* sp. isolated from poultry products in the state of Ceara, Brazil. Braz. J. Poult. Sci..

[35] Duarte D.A.M., Ribeiro A.R., Vasconcelos A.M.M., Santos S.B., Silva J.V.D., de Andrade P.L.A., da Costa de Arruda Falcão L.S.P. (2009). Occurrence of *Salmonella* spp. in broiler chicken carcasses and their susceptibility to antimicrobial agents. Braz. J. Microbiol..

[36] Penha Filho R.A.C., Ferreira J.C., Kanashiro A.M.I., Berchieri A., Darini A.L.C. (2019). Emergent multidrug-resistant nontyphoidal Salmonella serovars isolated from poultry in Brazil coharboring blaCTX-M-2 and qnrB or blaCMY-2 in large plasmids. Diagn. Microbiol. Infect. Dis..

[37] Vaz C.S.L., Streck A.F., Michael G.B., Marks F.S., Rodrigues D.P., dos Reis E.M.F., Cardoso M.R., Canal C.W. (2010). Antimicrobial resistance and subtyping of *Salmonella enterica* subspecies *enterica* serovar *Enteritidis* isolated from human outbreaks and poultry in southern Brazil. Poult. Sci..

[38] Medeiros M.A.N., de Oliveira D.C.N., Rodrigues D.P., de Freitas D.R.C. (2011). Prevalence and antimicrobial resistance of Salmonella in chicken carcasses at retail in 15 Brazilian cities. Rev. Panam. Salud Publica Pan Am. J. Public Health.

[39] Kottwitz L.B.M., Scheffer M.C., Costa L.M.D., Leitao J.A., Back A., Rodrigues D.P., Magnani M., de Oliveira T.C.R.M. (2012). Perfil de resistência a antimicrobianos, fagotipagem e caracterização molecular de cepas de *Salmonella enteritidis* de origem avícola. Semin. Ciênc. Agrár..

[40] Kottwitz L.B.M., Leão J.A., Back A., Rodrigues D.P., Magnani M., de Oliveira T.C.R.M. (2013). Commercially laid eggs vs. discarded hatching eggs: Contamination by *Salmonella* spp.. Braz. J. Microbiol. Publ. Braz. Soc. Microbiol..

[41] Campioni F., Zoldan M.M., Falcão J.P. (2014). Characterization of *Salmonella enteritidis* strains isolated from poultry and farm environments in Brazil. Epidemiol. Infect..

[42] Pandini J.A., da Pinto F.G.S., Muller J.M., Weber L.D., de Moura A.C. (2015). Ocorrência e perfil de resistencia antimicrobiana de sorotipos de *Salmonella* spp. isolados de aviários do Paraná, Brasil. Arq. Inst. Biol..

[43] Palmeira A., dos Santos L.R., Borsoi A., Rodrigues L.B., Calasans M., do Nascimento V.P. (2016). Serovars and antimicrobial resistance of *Salmonella* spp. isolated from turkey and broiler carcasses in southern Brazil between 2004 and 2006. Rev. Inst. Med. Trop. São Paulo.

[44] Borges K.A., Furian T.Q., de Souza S.N., Menezes R., Salle C.T.P., de Souza Moraes H.L., Tondo E.C., do Nascimento V.P. (2017). Phenotypic and molecular characterization of *Salmonella enteritidis* SE86 isolated from poultry and Salmonellosis outbreaks. Foodborne Pathog. Dis..

[45] Koerich P.K.V., Fonseca B.B., Balestrin E., Tagliari V., Hoepers P.G., Ueira-Vieira C., Oldoni I., Rauber R.H., Ruschel L., Nascimento V.P. (2018). *Salmonella gallinarum* field isolates and its relationship to vaccine strain SG9R. Br. Poult. Sci..

[46] Jajere S.M. (2019). A review of *Salmonella enterica* with particular focus on the pathogenicity and virulence factors, host specificity and antimicrobial resistance including multidrug resistance. Vet. World.

[47] Baptista D.Q., Santos A.F.M., Aquino M.H.C., Abreu D.L.C., Rodrigues D.P., Nascimento E.R., Pereira V.L.A. (2018). Prevalência e susceptibilidade antimicrobiana de sorotipos de *Salmonella* spp. isolados de frangos vivos e carcaças no estado do Rio de Janeiro. Pesqui. Vet. Bras..

[48] Public Health Agency of Canadá Salmonella Heidelberg—Ceftiofur-Related Resistance in Human and Retail Chicken Isolates. https://www.canada.ca/en/health-canada/services/drugs-health-products/veterinary-drugs/antimicrobial-resistance/notice-stakeholders-collaborative-efforts-promote-judicious-use-medically-important-antimicrobial-drugs-food-animal-production.html.

[49] Webber B., Borges K.A., Furian T.Q., Rizzo N.N., Tondo E.C., dos Santos L.R., Rodrigues L.B., do Nascimento V.P., Webber B., Borges K.A. (2019). Detection of virulence genes in Salmonella Heidelberg isolated from chicken carcasses. Rev. Inst. Med. Trop. São Paulo.

[50] Voss-Rech D., Vaz C.S.L., Alves L., Coldebella A., Leão J.A., Rodrigues D.P., Back A. (2015). A temporal study of *Salmonella enterica* serotypes from broiler farms in Brazil. Poult. Sci..

[51] Voss-Rech D., Kramer B., Silva V.S., Rebelatto R., Abreu P.G., Coldebella A., Vaz C.S.L. (2019). Longitudinal study reveals persistent environmental Salmonella Heidelberg in Brazilian broiler farms. Vet. Microbiol..

[52] Costa R.G., Festivo M.L., Araujo M.S., Reis E.M.F., Lázaro N.S., Rodrigues D.P. (2013). Antimicrobial susceptibility and serovars of salmonella circulating in commercial poultry carcasses and poultry products in Brazil. J. Food Prot..

[53] Voss-Rech D., Potter L., Vaz C.S.L., Pereira D.I.B., Sangioni L.A., Vargas Á.C., de Avila Botton S. (2017). Antimicrobial resistance in nontyphoidal Salmonella isolated from human and poultry-related samples in Brazil: 20-Year meta-analysis. Foodborne Pathog. Dis..

[54] da Cunha-Neto A., Carvalho L.A., Carvalho R.C.T., dos Prazeres Rodrigues D., Mano S.B., de Figueiredo E.E.S., Conte C.A. (2018). Salmonella isolated from chicken carcasses from a slaughterhouse in the state of Mato Grosso, Brazil: Antibiotic resistance profile, serotyping, and characterization by repetitive sequence-based PCR system. Poult. Sci..

[55] Perin A.P., Martins B.T.F., Barreiros M.A.B., Yamatogi R.S., Nero L.A., Dos Santos Bersot L. (2020). Occurrence, quantification, pulse types, and antimicrobial susceptibility of *Salmonella* sp. isolated from chicken meat in the state of Paraná, Brazil. Braz. J. Microbiol. Publ. Braz. Soc. Microbiol..

[56] Borges K.A., Furian T.Q., Souza S.N., Salle C.T.P., Moraes H.L.S., Nascimento V.P., Borges K.A., Furian T.Q., Souza S.N., Salle C.T.P. (2019). Antimicrobial resistance and molecular characterization of *Salmonella enterica* serotypes isolated from poultry sources in Brazil. Braz. J. Poult. Sci..

[57] Monte D.F., Lincopan N., Berman H., Cerdeira L., Keelara S., Thakur S., Fedorka-Cray P.J., Landgraf M. (2019). Genomic features of high-priority *Salmonella enterica* serovars circulating in the food production chain, Brazil, 2000–2016. Sci. Rep..

[58] Gazal L.E.S., Puño-Sarmiento J.J., Medeiros L.P., Cyoia P.S., da Silveira W.D., Kobayashi R.K.T., Nakazato G. (2015). Presence of pathogenicity islands and virulence genes of extraintestinal pathogenic *Escherichia coli* (ExPEC) in isolates from avian organic fertilizer. Poult. Sci..

[59] Bezerra W.G.A., da Silva I.N.G., Vasconcelos R.H., Machado D.N., Lopes E.D.S., Lima S.V.G., de Teixeira R.S.C., Lima J.B., Oliveira F.R., Maciel W.C. (2016). Isolation and antimicrobial resistance of *Escherichia coli* and *Salmonella enterica* subsp. *enterica* (O:6,8) in broiler chickens. Acta Sci. Vet..

[60] Maciel J.F., Matter L.B., Trindade M.M., Camillo G., Lovato M., de Ávila Botton S., Castagna de Vargas A. (2017). Virulence factors and antimicrobial susceptibility profile of extraintestinal *Escherichia coli* isolated from an avian colisepticemia outbreak. Microb. Pathog..

[61] Stella A.E., Oliveira M.C.D., da Fontana V.L.D.S., Maluta R.P., Borges C.A., de Ávila F.A. (2016). Characterization and antimicrobial resistance patterns of *Escherichia coli* isolated from feces of healthy broiler chickens. Arq. Inst. Biol..

[62] Borzi M.M., Cardozo M.V., de Oliveira E.S., de Pollo A.S., Guastalli E.A.L., dos Santos L.F., de Ávila F.A. (2018). Characterization of avian pathogenic *Escherichia coli* isolated from free-range helmeted guineafowl. Braz. J. Microbiol..

[63] Lima-Filho J.V., Martins L.V., de Nascimento D.C.O., Ventura R.F., Batista J.E.C., Silva A.F.B., Ralph M.T., Vaz R.V., Rabello C.B.-V., de Matos Mendes da Silva I. (2013). Zoonotic potential of multidrug-resistant extraintestinal pathogenic *Escherichia coli* obtained from healthy poultry carcasses in Salvador, Brazil. Braz. J. Infect. Dis..

[64] Ferro I.D., Benetti T.M., Oliveira T.C.R.M., Abrahão W.M., Farah S.M.S.S., Luciano F.B., Macedo R.E.F. (2015). Evaluation of antimicrobial resistance of *Campylobacter* spp. isolated from broiler carcasses. Br. Poult. Sci..

[65] Braga J.F.V., Chanteloup N.K., Trotereau A., Baucheron S., Guabiraba R., Ecco R., Schouler C. (2016). Diversity of *Escherichia coli* strains involved in vertebral osteomyelitis and arthritis in broilers in Brazil. BMC Vet. Res..

[66] Carvalho D., Finkler F., Grassotti T.T., Kunert Filho H.C., de Lima F.E.S., Soares B.D., Rossato J.M., da Cunha A.C., de Brito K.C.T., de Brito B.G. (2015). Antimicrobial susceptibility and pathogenicity of *Escherichia coli* strains of environmental origin. Ciênc. Rural.

[67] Vaz R.V., Gouveia G.V., Andrade N.M.J., da Costa M.M., Lima-Filho J.V. (2017). Phylogenetic characterization of serum plus antibiotic-resistant extraintestinal *Escherichia coli* obtained from the liver of poultry carcasses in Pernambuco. Pesqui. Vet. Bras..

[68] Barros M.R. (2012). Resistência antimicrobiana e perfil plasmidial de *Escherichia coli* isolada de frangos de corte e poedeiras comerciais no estado de pernambuco. Pesq. Vet. Bras..

[69] Koga V.L., Scandorieiro S., Vespero E.C., Oba A., de Brito B.G., de Brito K.C.T., Nakazato G., Kobayashi R.K.T. (2015). Comparison of antibiotic resistance and virulence factors among *Escherichia coli* isolated from conventional and free-range poultry. BioMed Res. Int..

[70] De Moura Oliveira K.A., Mendonca R.C.S., De Oliveira G.V., Sodre A.F. (2006). Antibiotic resistance of Campylobacter isolated from automated broiler farms. J. Food Saf..

[71] Kuana S.L., dos Santos L.R., Rodrigues L.B., Borsoi A., do Moraes H.L.S., Salle C.T.P., do Nascimento V.P. (2008). Antimicrobial resistance in *Campylobacter* spp isolated from broiler flocks. Braz. J. Microbiol..

[72] Ku B.K., Kim H.J., Lee Y.J., Kim Y.I., Choi J.S., Park M.Y., Kwon J.W., Nam H.-M., Kim Y.H., Jung S.-C. (2011). Genetic characterization and antimicrobial susceptibility of *Campylobacter* spp. isolated from domestic and imported chicken meats and humans in Korea. Foodborne Pathog. Dis..

[73] Melo R.T., Grazziotin A.L., Júnior E.C.V., Prado R.R., Mendonça E.P., Monteiro G.P., Peres P.A.B.M., Rossi D.A. (2019). Evolution of Campylobacter jejuni of poultry origin in Brazil. Food Microbiol..

[74] Moraes D.M.C., Andrade M.A., Minafra-Rezende C.S., de Barnabé A.C.S., de Jayme V.S., Nunes I.A., de Batista D.A. (2014). Fontes de infecção e perfil de suscetibilidade aos antimicrobianos de *Salmonella* sp. isoladas no fluxo de produção de frangos de corte. Arq. Inst. Biol..

[75] Minharro S., Nascimento C.A., Galletti J.P., Merisse T.J., Feitosa A.C.F., Santos H.D., Dias F.E.F., Santana E.S., Baldani C.D., Andrade M.A. (2015). Antimicrobial susceptibility of Salmonella serovars isolated from edible offal and carcasses of slaughtered poultry in the state of Tocantins, Brazil. Semina Ciênc. Agrár..

[76] de Moura H.M., Silva P.R., da Silva P.H.C., Souza N.R., Racanicci A.M.C., Santana Â.P. (2013). Antimicrobial resistance of Campylobacter jejuni isolated from chicken carcasses in the federal district, Brazil. J. Food Prot..

[77] Brazil Ministério da Súde (MS) (2018). Surtos de Doenças Transmitidas por Alimentos no Brasil. https://portalarquivos2.saude.gov.br/images/pdf/2018/janeiro/17/Apresentacao-Surtos-DTA-2018.pdf.

[78] European Food Safety Authority (EFSA), European Centre for Disease Prevention and Control (ECDC) (2018). The European Union summary report on trends and sources of zoonoses, zoonotic agents and food-borne outbreaks in 2017. EFSA J..

[79] Arguello H., Álvarez-Ordoñez A., Carvajal A., Rubio P., Prieto M. (2013). Role of slaughtering in Salmonella spreading and control in pork production. J. Food Prot..

[80] Lopes G.V., Pissetti C., da Cruz Payão Pellegrini D., da Silva L.E., Cardoso M. (2015). Resistance phenotypes and genotypes of *Salmonella enterica* subsp. *enterica* isolates from feed, pigs, and carcasses in Brazil. J. Food Prot..

[81] Kich J.D., Coldebella A., Morés N., Nogueira M.G., Cardoso M., Fratamico P.M., Call J.E., Fedorka-Cray P., Luchansky J.B. (2011). Prevalence, distribution, and molecular characterization of Salmonella recovered from swine finishing herds and a slaughter facility in Santa Catarina, Brazil. Int. J. Food Microbiol..

[82] Centers for Disease Control and Prevention (CDC) Advice to Clinicians—Multistate Outbreak of Salmonella Infections Linked to Raw Chicken Products, October 2018. https://www.cdc.gov/salmonella/infantis-10-18/advice.html.

[83] Almeida F., Medeiros M.I.C., Kich J.D., Falcão J.P. (2016). Virulence-Associated genes, antimicrobial resistance and molecular typing of *Salmonella typhimurium* strains isolated from swine from 2000 to 2012 in Brazil. J. Appl. Microbiol..

[84] Souto M.S.M., Coura F.M., Dorneles E.M.S., Stynen A.P.R., Alves T.M., Santana J.A., Pauletti R.B., Guedes R.M.C., Viott A.M., Heinemann M.B. (2017). Antimicrobial susceptibility and phylotyping profile of pathogenic *Escherichia coli* and *Salmonella enterica* isolates from calves and pigs in Minas Gerais, Brazil. Trop. Anim. Health Prod..

[85] Viana C., Sereno M.J., Pegoraro K., Yamatogi R.S., Call D.R., dos Santos Bersot L., Nero L.A. (2019). Distribution, diversity, virulence genotypes and antibiotic resistance for Salmonella isolated from a Brazilian pork production chain. Int. J. Food Microbiol..

[86] Spindola M.G., Cunha M.P.V., Moreno L.Z., Amigo C.R., Silva A.P.S., Parra B.M., Poor A.P., de Oliveira C.H., Perez B.P., Knöbl T. (2018). Genetic diversity, virulence genotype and antimicrobial resistance of uropathogenic *Escherichia coli* (UPEC) isolated from sows. Vet. Q..

[87] Silva K.C., Moreno M., Cabrera C., Spira B., Cerdeira L., Lincopan N., Moreno A.M. (2016). First characterization of CTX-M-15-producing *Escherichia coli* strains belonging to sequence type (ST) 410, ST224, and ST1284 from commercial swine in South America. Antimicrob. Agents Chemother..

[88] Morales A.S., Fragoso de Araújo J., de Moura Gomes V.T., Reis Costa A.T., dos Prazeres Rodrigues D., Porfida Ferreira T.S., de Lima Filsner P.H.N., Felizardo M.R., Micke Moreno A. (2012). Colistin resistance in *Escherichia coli* and *Salmonella enterica* strains isolated from swine in Brazil. Sci. World J..

[89] Kieffer N., Nordmann P., Moreno A.M., Zanolli Moreno L., Chaby R., Breton A., Tissières P., Poirel L. (2018). Genetic and functional characterization of an MCR-3-Like enzyme-producing *Escherichia coli* isolate recovered from swine in Brazil. Antimicrob. Agents Chemother..

[90] Van Damme I., Berkvens D., Vanantwerpen G., Baré J., Houf K., Wauters G., De Zutter L. (2015). Contamination of freshly slaughtered pig carcasses with enteropathogenic *Yersinia* spp.: Distribution, quantification and identification of risk factors. Int. J. Food Microbiol..

[91] Martins B.T.F., Botelho C.V., Silva D.A.L., Lanna F.G.P.A., Grossi J.L., Campos-Galvão M.E.M., Yamatogi R.S., Falcão J.P., dos Bersot L.S., Nero L.A. (2018). *Yersinia enterocolitica* in a Brazilian pork production chain: Tracking of contamination routes, virulence and antimicrobial resistance. Int. J. Food Microbiol..

[92] Rusak L.A., Dos Reis C.M.F., Barbosa A.V., Santos A.F.M., Paixão R., Hofer E., Vallim D.C., Asensi M.D. (2014). Phenotypic and genotypic analysis of bio-serotypes of *Yersinia enterocolitica* from various sources in Brazil. J. Infect. Dev. Ctries..

[93] Frazão M.R., Andrade L.N., Darini A.L.C., Falcão J.P. (2017). Antimicrobial resistance and plasmid replicons in *Yersinia enterocolitica* strains isolated in Brazil in 30 years. Braz. J. Infect. Dis..

[94] Fàbrega A., Roca I., Vila J. (2010). Fluoroquinolone and multidrug resistance phenotypes associated with the overexpression of AcrAB and an orthologue of MarA in *Yersinia enterocolitica*. Int. J. Med. Microbiol..

[95] Rau R.B., de Lima-Morales D., Wink P.L., Ribeiro A.R., Martins A.F., Barth A.L. (2018). Emergence of *mcr-*1 producing *Salmonella enterica* serovar *Typhimurium* from retail meat: First detection in Brazil. Foodborne Pathog. Dis..

[96] Brazil Companhia Nacional de Abastecimento (CONAB) (2018). Compêndio de Estudos da Conab-v16—Pecuária Leiteira—Análise dos Custos. https://www.conab.gov.br/institucional/publicacoes/compendio-de-estudos-da-conab.

[97] Sindicado Nacional da Indústria de Produtos para Saúde Animal (SINDAM) Compêndio de Produtos Veterinários. https://sistemas.sindan.org.br/cpvs/.

[98] Keane O.M. (2019). Symposium review: Intramammary infections—Major pathogens and strain-associated complexity. J. Dairy Sci..

[99] Silva N.C.C., Guimarães F.F., Manzi M.P., Júnior A.F., Gómez-Sanz E., Gómez P., Langoni H., Rall V.L.M., Torres C. (2014). Methicillin-Resistant *Staphylococcus aureus* of lineage ST398 as cause of mastitis in cows. Lett. Appl. Microbiol..

[100] da Costa Krewer C., Santos Amanso E., Veneroni Gouveia G., de Lima Souza R., da Costa M.M., Aparecido Mota R. (2015). Resistance to antimicrobials and biofilm formation in *Staphylococcus* spp. isolated from bovine mastitis in the Northeast of Brazil. Trop. Anim. Health Prod..

[101] Bonsaglia E.C.R., Silva N.C.C., Rossi B.F., Camargo C.H., Dantas S.T.A., Langoni H., Guimarães F.F., Lima F.S., Fitzgerald J.R., Fernandes A. (2018). Molecular epidemiology of methicillin-susceptible *Staphylococcus aureus* (MSSA) isolated from milk of cows with subclinical mastitis. Microb. Pathog..

[102] Lange C., Cardoso M., Senczek D., Schwarz S. (1999). Molecular subtyping of *Staphylococcus aureus* isolates from cases of bovine mastitis in Brazil. Vet. Microbiol..

[103] Cabral K.G., Lämmler C., Zschöck M., Langoni H., de Sá M.E.P., Victória C., Da Silva A.V. (2004). Pheno and genotyping of *Staphylococcus aureus*, isolated from bovine milk samples from São Paulo State, Brazil. Can. J. Microbiol..

[104] Rabello R.F., Souza C.R.V.M., Duarte R.S., Lopes R.M.M., Teixeira L.M., Castro A.C.D. (2005). Characterization of *Staphylococcus aureus* isolates recovered from bovine mastitis in Rio de Janeiro, Brazil. J. Dairy Sci..

[105] Aires-de-Sousa M., Parente C.E.S.R., Vieira-da-Motta O., Bonna I.C.F., Silva D.A., de Lencastre H. (2007). Characterization of *Staphylococcus aureus* isolates from buffalo, bovine, ovine, and caprine milk samples collected in Rio de Janeiro State, Brazil. Appl. Environ. Microbiol..

[106] Ceotto H., dos Nascimento J.S., de Paiva Brito M.A.V., do Carmo de Freire Bastos M. (2009). Bacteriocin production by *Staphylococcus aureus* involved in bovine mastitis in Brazil. Res. Microbiol..

[107] Laport M.S., Marinho P.R., da Santos O.C.S., de Almeida P., Romanos M.T.V., Muricy G., Brito M.A.V.P., Giambiagi-deMarval M. (2012). Antimicrobial activity of marine sponges against coagulase-negative staphylococci isolated from bovine mastitis. Vet. Microbiol..

[108] Martini C.L., Lange C.C., Brito M.A., Ribeiro J.B., Mendonça L.C., Vaz E.K. (2017). Characterisation of penicillin and tetracycline resistance in *Staphylococcus aureus* isolated from bovine milk samples in Minas Gerais, Brazil. J. Dairy Res..

[109] Fernandes dos Santos F., Mendonça L.C., de Reis D.R.L., de Guimarães A.S., Lange C.C., Ribeiro J.B., Machado M.A., Brito M.A.V.P. (2016). Presence of mecA-positive multidrug-resistant *Staphylococcus epidermidis* in bovine milk samples in Brazil. J. Dairy Sci..

[110] Marques V.F., da Motta C.C., da Soares B.S., de Melo D.A., Coelho S.M., da Silva Coelho I., Barbosa H., Souza M.S. (2017). Biofilm production and beta-lactamic resistance in Brazilian *Staphylococcus aureus* isolates from bovine mastitis. Braz. J. Microbiol..

[111] Castelani L., Pilon L.E., Martins T., Pozzi C.R., Arcaro J.R.P. (2014). Investigation of biofilm production and *icaA* and *icaD* genes in *Staphylococcus aureus* isolated from heifers and cows with mastitis: Mastitis. Anim. Sci. J..

[112] Haubert L., Kroning I.S., Iglesias M.A., da Silva W.P. (2017). First report of the *Staphylococcus aureus* isolate from subclinical bovine mastitis in the South of Brazil harboring resistance gene dfrG and transposon family Tn 916-1545. Microb. Pathog..

[113] Freitas C.H., Mendes J.F., Villarreal P.V., Santos P.R., Gonçalves C.L., Gonzales H.L., Nascente P.S. (2018). Identification and antimicrobial suceptibility profile of bacteria causing bovine mastitis from dairy farms in Pelotas, Rio Grande do Sul. Braz. J. Biol..

[114] Costa G.M., Paiva L.V., Figueiredo H.C.P., Figueira A.R., Pereira U.P., Silva N. (2012). Population diversity of *Staphylococcus aureus* isolated from bovine mastitis in Brazilian dairy herds. Res. Vet. Sci..

[115] Otto M. (2013). Community-Associated MRSA: What makes them special?. Int. J. Med. Microbiol..

[116] Bassetti M., Carnelutti A., Castaldo N., Peghin M. (2019). Important new therapies for methicillin-resistant *Staphylococcus aureus*. Expert Opin. Pharmacother..

[117] Silva N.C.C., Guimarães F.F., Manzi M.P., Budri P.E., Gómez-Sanz E., Benito D., Langoni H., Rall V.L.M., Torres C. (2013). Molecular characterization and clonal diversity of methicillin-susceptible *Staphylococcus aureus* in milk of cows with mastitis in Brazil. J. Dairy Sci..

[118] Mello P.L., Pinheiro L., de Martins L.A., Brito M.A.V.P., de Lourdes Ribeiro de Souza da Cunha M. (2017). Short communication: β-Lactam resistance and vancomycin heteroresistance in *Staphylococcus* spp. isolated from bovine subclinical mastitis. J. Dairy Sci..

[119] Guimarães F.F., Manzi M.P., Joaquim S.F., Richini-Pereira V.B., Langoni H. (2017). Short communication: Outbreak of methicillin-resistant *Staphylococcus aureus* (MRSA)-associated mastitis in a closed dairy herd. J. Dairy Sci..

[120] Miranda P.S.D., Lannes-Costa P.S., Pimentel B.A.S., Silva L.G., Ferreira-Carvalho B.T., Menezes G.C., Mattos-Guaraldi A.L., Hirata R., Mota R.A., Nagao P.E. (2018). Biofilm formation on different pH conditions by *Streptococcus agalactiae* isolated from bovine mastitic milk. Lett. Appl. Microbiol..

[121] Pinto T.C.A., Costa N.S., Vianna Souza A.R., da Silva L.G., de Corrêa A.B.A., Fernandes F.G., Oliveira I.C.M., de Mattos M.C., Rosado A.S., Benchetrit L.C. (2013). Distribution of serotypes and evaluation of antimicrobial susceptibility among human and bovine *Streptococcus agalactiae* strains isolated in Brazil between 1980 and 2006. Braz. J. Infect. Dis..

[122] Fernandes J.B.C., Zanardo L.G., Galvão N.N., Carvalho I.A., Nero L.A., Moreira M.A.S. (2011). *Escherichia coli* from clinical mastitis: Serotypes and virulence factors. J. Vet. Diagn. Invest..

[123] Zanella G.N., Mikcha J.M.G., Bando E., Siqueira V.L.D., Machinski M. (2010). Occurrence and antibiotic resistance of coliform bacteria and antimicrobial residues in pasteurized cow’s milk from Brazil. J. Food Prot..

[124] dos Alves T.S., Lara G.H.B., Maluta R.P., Ribeiro M.G., da Leite D.S. (2018). Carrier flies of multidrug-resistant *Escherichia coli* as potential dissemination agent in dairy farm environment. Sci. Total Environ..

[125] United States of America Department of Agriculture (USDA), Economic Research Service (ERS) Brazil Once Again Becomes the World’s Largest Beef Exporter. https://www.ers.usda.gov/amber-waves/2019/july/brazil-once-again-becomes-the-world-s-largest-beef-exporter.

[126] dos Santos E.C.C., Castro V.S., Cunha-Neto A., dos Santos L.F., Vallim D.C., de Lisbôa R.C., Carvalho R.C.T., Junior C.A.C., de Figueiredo E.E.S. (2018). *Escherichia coli* O26 and O113:H21 on carcasses and beef from a slaughterhouse located in Mato Grosso, Brazil. Foodborne Pathog. Dis..

[127] Fernandes F.P., Voloski F.L.S., Ramires T., Haubert L., Reta G.G., Mondadori R.G., da Silva W.P., de Cássia dos Santos da Conceição R., Duval E.H. (2017). Virulence and antimicrobial resistance of *Salmonella* spp. and *Escherichia coli* in the beef jerky production line. FEMS Microbiol. Lett..

[128] Loiko M.R., de Paula C.M.D., Langone A.C.J., Rodrigues R.Q., Cibulski S., de Rodrigues R.O., Camargo A.C., Nero L.A., Mayer F.Q., Tondo E.C. (2016). Genotypic and antimicrobial characterization of pathogenic bacteria at different stages of cattle slaughtering in southern Brazil. Meat Sci..

[129] Cossi M.V.C., Burin R.C.K., Lopes D.A., Dias M.R., de Castilho N.P.A., de Arruda Pinto P.S., Nero L.A. (2013). Antimicrobial resistance and virulence profiles of Salmonella isolated from butcher shops in Minas Gerais, Brazil. J. Food Prot..

[130] da Silva F.F.P., Horvath M.B., Silveira J.G., Pieta L., Tondo E.C. (2014). Occurrence of *Salmonella* spp. and generic *Escherichia coli* on beef carcasses sampled at a Brazilian slaughterhouse. Braz. J. Microbiol..

[131] Camargo A.C., de Castilho N.P.A., da Silva D.A.L., Vallim D.C., Hofer E., Nero L.A. (2015). Antibiotic resistance of *Listeria monocytogenes* isolated from meat-processing environments, beef products, and clinical cases in Brazil. Microb. Drug Resist..

[132] Silva N.C.C., Guimarães F.F., de P. Manzi M., Gómez-Sanz E., Gómez P., Araújo J.P., Langoni H., Rall V.L.M., Torres C. (2014). Characterization of methicillin-resistant coagulase-negative Staphylococci in milk from cows with mastitis in Brazil. Antonie Van Leeuwenhoek.

[133] Camargo A.C., Lafisca A., Cossi M.V.C., Lanna F.G.P.A., Dias M.R., de Arruda Pinto P.S., Nero L.A. (2014). Low occurrence of *Listeria monocytogenes* on bovine hides and carcasses in Minas Gerais State, Brazil: Molecular characterization and antimicrobial resistance. J. Food Prot..

[134] Warren R.E., Ensor V.M., O’Neill P., Butler V., Taylor J., Nye K., Harvey M., Livermore D.M., Woodford N., Hawkey P.M. (2008). Imported chicken meat as a potential source of quinolone-resistant *Escherichia coli* producing extended-spectrum beta-lactamases in the UK. J. Antimicrob. Chemother..

[135] Dhanji H., Murphy N.M., Doumith M., Durmus S., Surman Lee S., Hope R., Woodford N., Livermore D.M. (2010). Cephalosporin resistance mechanisms in *Escherichia coli* isolated from raw chicken imported into the UK. J. Antimicrob. Chemother..

[136] Casella T., Rodríguez M.M., Takahashi J.T., Ghiglione B., Dropa M., Assunção E., Nogueira M.L., Lincopan N., Gutkind G., Nogueira M.C.L. (2015). Detection of blaCTX-M-type genes in complex class 1 integrons carried by Enterobacteriaceae isolated from retail chicken meat in Brazil. Int. J. Food Microbiol..

[137] Ferreira J.C., Penha Filho R.A.C., Andrade L.N., Berchieri A., Darini A.L.C. (2016). Evaluation and characterization of plasmids carrying CTX-M genes in a non-clonal population of multidrug-resistant Enterobacteriaceae isolated from poultry in Brazil. Diagn. Microbiol. Infect. Dis..

[138] Moura Q., Fernandes M.R., Silva K.C., Monte D.F., Esposito F., Dropa M., Noronha C., Moreno A.M., Landgraf M., Negrão F.J. (2018). Virulent nontyphoidal Salmonella producing CTX-M and CMY-2 β-lactamases from livestock, food and human infection, Brazil. Virulence.

[139] Galetti R., Antonio Casarin Penha Filho R., Ferreira J.C., Varani M.A., Costa Darini A.L. (2019). Antibiotic resistance and heavy metal tolerance plasmids: The antimicrobial bulletproof properties of *Escherichia fergusonii* isolated from poultry. Infect. Drug Resist..

[140] Botelho L.A.B., Kraychete G.B., Costa e Silva J.L., Regis D.V.V., Picão R.C., Moreira B.M., Bonelli R.R. (2015). Widespread distribution of CTX-M and plasmid-mediated AmpC β-lactamases in *Escherichia coli* from Brazilian chicken meat. Mem. Inst. Oswaldo Cruz.

[141] Fitch F.M., Carmo-Rodrigues M.S., Oliveira V.G.S., Gaspari M.V., dos Santos A., de Freitas J.B., Pignatari A.C.C. (2016). β-Lactam resistance genes: Characterization, epidemiology, and first detection of *bla*_CTX-M-1_ and *bla*_CTX-M-14_ in *Salmonella* spp. isolated from poultry in Brazil—Brazil Ministry of Agriculture’s Pathogen Reduction Program. Microb. Drug Resist..

[142] Ibbe R., Ferreira K.F.S., Silva R.L., Machado S.A., Nascimento E.R., Rodrigues D.P., Aquino M.H.C., de Almeida Pereira V.L. (2017). Amoxicillin/Clavulanic acid and cefotaxime resistance in Salmonella Minnesota and Salmonella Heidelberg from broiler chickens. Poult. Sci. J..

[143] Ferreira J.C., Penha Filho R.A.C., Andrade L.N., Berchieri A., Darini A.L.C. (2017). Diversity of plasmids harboring *bla*_CMY-2_ in multidrug-resistant *Escherichia coli* isolated from poultry in Brazil. Diagn. Microbiol. Infect. Dis..

[144] Tiba-Casas M.R., Camargo C.H., Soares F.B., Doi Y., Fernandes S.A. (2019). Emergence of CMY-2-Producing Salmonella Heidelberg associated with IncI1 plasmids isolated from poultry in Brazil. Microb. Drug Resist..

[145] Fernandes S.A., Camargo C.H., Francisco G.R., Bueno M.F.C., Garcia D.O., Doi Y., Casas M.R.T. (2017). Prevalence of extended-spectrum β-lactamases CTX-M-8 and CTX-M-2-producing Salmonella serotypes from clinical and nonhuman isolates in Brazil. Microb. Drug Resist..

[146] Casella T., Haenni M., Madela N.K., de Andrade L.K., Pradela L.K., de Andrade L.N., da Darini A.L.C., Madec J.-Y., Nogueira M.C.L. (2018). Extended-Spectrum cephalosporin-resistant *Escherichia coli* isolated from chickens and chicken meat in Brazil is associated with rare and complex resistance plasmids and pandemic ST lineages. J. Antimicrob. Chemother..

[147] Cunha M.P.V., Lincopan N., Cerdeira L., Esposito F., Dropa M., Franco L.S., Moreno A.M., Knöbl T. (2017). Coexistence of CTX-M-2, CTX-M-55, CMY-2, FosA3, and QnrB19 in extraintestinal pathogenic *Escherichia coli* from poultry in Brazil. Antimicrob. Agents Chemother..

[148] Koga V.L., Rodrigues G.R., Scandorieiro S., Vespero E.C., Oba A., de Brito B.G., de Brito K.C.T., Nakazato G., Kobayashi R.K.T. (2015). Evaluation of the antibiotic resistance and virulence of *Escherichia coli* strains isolated from chicken carcasses in 2007 and 2013 from Paraná, Brazil. Foodborne Pathog. Dis..

[149] Cyoia P.S., Koga V.L., Nishio E.K., Houle S., Dozois C.M., de Brito K.C.T., de Brito B.G., Nakazato G., Kobayashi R.K.T. (2019). Distribution of ExPEC virulence factors, *bla*_CTX-M_, *fos*A3, and *mcr*-1 in *Escherichia coli* isolated from commercialized chicken carcasses. Front. Microbiol..

[150] Zogg A.L., Zurfluh K., Nüesch-Inderbinen M., Stephan R. (2016). Characteristics of ESBL-producing Enterobacteriaceae and Methicillinresistant *Staphylococcus aureus* (MRSA) isolated from Swiss and imported raw poultry meat collected at retail level. Schweiz. Arch. Tierheilkd..

[151] Nahar A., Awasthi S.P., Hatanaka N., Okuno K., Hoang P.H., Hassan J., Hinenoya A., Yamasaki S. (2018). Prevalence and characteristics of extended-spectrum β-lactamase-producing *Escherichia coli* in domestic and imported chicken meats in Japan. J. Vet. Med. Sci..

[152] Kim Y.-J., Moon J.-S., Oh D.-H., Chon J.-W., Song B.-R., Lim J.-S., Heo E.-J., Park H.-J., Wee S.-H., Sung K. (2018). Genotypic characterization of ESBL-producing *E. coli* from imported meat in South Korea. Food Res. Int..

[153] Hoepers P.G., Silva P.L., Rossi D.A., Valadares E.C., Ferreira B.C., Zuffo J.P., Koerich P.K., Fonseca B.B. (2018). The association between extended spectrum beta-lactamase (ESBL) and ampicillin C (AmpC) beta-lactamase genes with multidrug resistance in *Escherichia coli* isolates recovered from turkeys in Brazil. Br. Poult. Sci..

[154] Saraiva M.M.S., Moreira Filho A.L.B., Freitas Neto O.C., Silva N.M.V., Givisiez P.E.N., Gebreyes W.A., Oliveira C.J.B. (2018). Off-Label use of ceftiofur in one-day chicks triggers a short-term increase of ESBL-producing *E. coli* in the gut. PLoS ONE.

[155] Fernandes S.A., Paterson D.L., Ghilardi-Rodrigues Â.C., Adams-Haduch J.M., Tavechio A.T., Doi Y. (2009). CTX-M-2–producing *Salmonella typhimurium* isolated from pediatric patients and poultry in Brazil. Microb. Drug Resist..

[156] Moura Q., Fernandes M.R., Cerdeira L., Ienne S., Souza T.A., Negrão F.J., Lincopan N. (2017). Draft genome sequence of a multidrug-resistant CMY-2-producing *Salmonella enterica* subsp. *enterica* serovar Minnesota ST3088 isolated from chicken meat. J. Glob. Antimicrob. Resist..

[157] Botelho L.A.B., Kraychete G.B., Rocha P.B., da Silva A.P.S., Picão R.C., Moreira B.M., Bonelli R.R. (2020). CTX-M-and pAmpC-encoding genes are associated with similar mobile genetic elements in *Escherichia coli* isolated from different brands of Brazilian chicken meat. Microb. Drug Resist..

[158] Martins P.D., de Almeida T.T., Basso A.P., de Moura T.M., Frazzon J., Tondo E.C., Frazzon A.P.G. (2013). Coagulase-Positive Staphylococci isolated from chicken meat: Pathogenic potential and vancomycin resistance. Foodborne Pathog. Dis..

[159] Rodrigues M.X., Silva N.C.C., Trevilin J.H., Cruzado M.M.B., Mui T.S., Duarte F.R.S., Castillo C.J.C., Canniatti-Brazaca S.G., Porto E. (2017). Molecular characterization and antibiotic resistance of *Staphylococcus* spp. isolated from cheese processing plants. J. Dairy Sci..

[160] Moreno L.Z., Dutra M.C., Moreno M., Ferreira T.S., da Silva G.F., Matajira C.E., Silva A.P.S., Moreno A.M. (2016). Vancomycin-Intermediate livestock-associated methicillin-resistant *Staphylococcus aureus* ST398/t9538 from swine in Brazil. Mem. Inst. Oswaldo Cruz.

[161] Xavier D.B., Bernal F.E.M., Titze-de-Almeida R. (2006). Absence of VanA-and VanB-containing Enterococci in poultry raised on nonintensive production farms in Brazil. Appl. Environ. Microbiol..

[162] Moraes P.M., Perin L.M., Todorov S.D., Silva A., Franco B.D.G.M., Nero L.A. (2012). Bacteriocinogenic and virulence potential of Enterococcus isolates obtained from raw milk and cheese. J. Appl. Microbiol..

[163] Perin L.M., Miranda R.O., Todorov S.D., de Franco B.D.G.M., Nero L.A. (2014). Virulence, antibiotic resistance and biogenic amines of bacteriocinogenic lactococci and Enterococci isolated from goat milk. Int. J. Food Microbiol..

[164] Sacramento A.G., Zanella R.C., de Almeida L.M., Pires C., Popazoglo C., Costa E.A.S., Cerdeira L.T., Mamizuka E.M., Lincopan N. (2016). Identification of new sequence types among *Enterococcus faecium* and *Enterococcus faecalis* carrying the vanA gene in retail chicken meat. J. Glob. Antimicrob. Resist..

[165] Fernandes M.R., Moura Q., Sartori L., Silva K.C., Cunha M.P., Esposito F., Lopes R., Otutumi L.K., Gonçalves D.D., Dropa M. (2016). Silent dissemination of colistin-resistant *Escherichia coli* in South America could contribute to the global spread of the *mcr*-1 gene. Eurosurveillance.

[166] Lentz S.A., de Lima-Morales D., Cuppertino V.M., de Nunes L.S., da Motta A.S., Zavascki A.P., Barth A.L., Martins A.F. (2016). Letter to the editor: *Escherichia coli* harbouring *mcr*-1 gene isolated from poultry not exposed to polymyxins in Brazil. Eurosurveillance.

[167] Fernandes M.R., Moura Q., Esposito F., Lincopan N. (2016). Authors’ reply: *Escherichia coli* harbouring *mcr-1* gene isolated from poultry not exposed to polymyxins in Brazil. Eurosurveillance.

[168] Oliveira C.C., Lopes E.S., Barbosa D.R., Pimenta R.L., Sobrinho N.M.B.A., Coelho S.M.O., Souza M.M.S., Coelho I.S. (2019). Occurrence of the colistin resistance *mcr*-1 gene in soils from intensive vegetable production and native vegetation. Eur. J. Soil Sci..

[169] Monte D.F., Mem A., Fernandes M.R., Cerdeira L., Esposito F., Galvão J.A., Franco B.D.G.M., Lincopan N., Landgraf M. (2017). Chicken meat as a reservoir of colistin-resistant *Escherichia coli* strains carrying *mcr-1* genes in South America. Antimicrob. Agents Chemother..

[170] Moreno L.Z., Gomes V.T.M., Moreira J., de Oliveira C.H., Peres B.P., Silva A.P.S., Thakur S., La Ragione R.M., Moreno A.M. (2019). First report of *mcr*-*1*-harboring *Salmonella enterica* serovar Schwarzengrund isolated from poultry meat in Brazil. Diagn. Microbiol. Infect. Dis..

[171] Aires C.A.M., da Conceição-Neto O.C., Tavares E., Oliveira T.R., Dias C.F., Montezzi L.F., Picão R.C., Albano R.M., Asensi M.D., Carvalho-Assef A.P.D. (2017). Emergence of the plasmid-mediated *mcr-1* gene in clinical KPC-2-producing *Klebsiella pneumoniae* sequence type 392 in Brazil. Antimicrob. Agents Chemother..

[172] Dalmolin T.V., Castro L., Mayer F.Q., Zavascki A.P., Martins A.F., de Lima-Morales D., Barth A.L. (2017). Co-Occurrence of *mcr-1* and *bla*_KPC-2_ in a clinical isolate of *Escherichia coli* in Brazil. J. Antimicrob. Chemother..

[173] Fernandes M.R., McCulloch J.A., Vianello M.A., Moura Q., Pérez-Chaparro P.J., Esposito F., Sartori L., Dropa M., Matté M.H., Lira D.P.A. (2016). First report of the globally disseminated IncX4 plasmid carrying the *mcr-1* gene in a colistin-resistant *Escherichia coli* sequence type 101 isolate from a human infection in Brazil. Antimicrob. Agents Chemother..

[174] Sellera F.P., Fernandes M.R., Sartori L., Carvalho M.P.N., Esposito F., Nascimento C.L., Dutra G.H.P., Mamizuka E.M., Pérez-Chaparro P.J., McCulloch J.A. (2017). *Escherichia coli* carrying IncX4 plasmid-mediated *mcr-1* and *bla*_CTX-M_ genes in infected migratory Magellanic penguins (*Spheniscus magellanicus*). J. Antimicrob. Chemother..

[175] Ferrari R., Galiana A., Cremades R., Rodríguez J.C., Magnani M., Tognim M.C.B., Oliveira T.C.R.M., Royo G. (2013). Plasmid-Mediated quinolone resistance (PMQR) and mutations in the topoisomerase genes of *Salmonella enterica* strains from Brazil. Braz. J. Microbiol..

[176] Campioni F., Souza R.A., Martins V.V., Stehling E.G., Bergamini A.M.M., Falcão J.P. (2017). Prevalence of *gyrA* mutations in nalidixic acid-resistant strains of *Salmonella enteritidis* isolated from humans, food, chickens, and the farm environment in Brazil. Microb. Drug Resist..

[177] Ferreira J.C., Penha Filho R.A.C., Kuaye A.P.Y., Andrade L.N., Berchieri A., da Darini A.L.C. (2018). Identification and characterization of plasmid-mediated quinolone resistance determinants in Enterobacteriaceae isolated from healthy poultry in Brazil. Infect. Genet. Evol..

[178] Ferreira J.C., Filho R.A.C.P., Andrade L.N., Berchieri A., Darini A.L.C. (2014). Detection of chromosomal *bla*_CTX-M-2_ in diverse *Escherichia coli* isolates from healthy broiler chickens. Clin. Microbiol. Infect..

[179] Brisola M.C., Crecencio R.B., Bitner D.S., Frigo A., Rampazzo L., Stefani L.M., Faria G.A. (2019). *Escherichia coli* used as a biomarker of antimicrobial resistance in pig farms of Southern Brazil. Sci. Total Environ..

[180] Panzenhagen P.H.N., Cabral C.C., Suffys P.N., Franco R.M., Rodrigues D.P., Conte C. (2018). A, Jr. Comparative genome analysis and characterization of the *Salmonella typhimurium* strain CCRJ_26 isolated from swine carcasses using whole-genome sequencing approach. Lett. Appl. Microbiol..

[181] Mattiello S.P., Drescher G., Barth V.C., Ferreira C.A.S., Oliveira S.D. (2015). Characterization of antimicrobial resistance in *Salmonella enterica* strains isolated from Brazilian poultry production. Antonie Van Leeuwenhoek.

[182] Van Boeckel T.P., Pires J., Silvester R., Zhao C., Song J., Criscuolo N.G., Gilbert M., Bonhoeffer S., Laxminarayan R. (2019). Global trends in antimicrobial resistance in animals in low-and middle-income countries. Science.

[183] Brazil, Ministério da Agricultura, Pecuária e Abastecimento (MAPA) Portaria no 396 de 23 de novembro de 2009. http://www.agricultura.gov.br/assuntos/inspecao/produtos-animal/plano-de-nacional-de-controle-de-residuos-e-contaminantes/documentos-da-pncrc/portaria-sda-n-o-396-de-23-de-novembro-de-2009.pdf/view.

[184] Brazil, Agência Nacional de Vigilância Sanitária (ANVISA) RDC no 54 de 12 de novembro de 2012. http://portal.anvisa.gov.br/documents/%2033880/2568070/rdc0054_12_11_2012.pdf/c5ac23fd-974e-4f2c-9fbc-48f7e0a31864.

[185] Food and Agriculture Organization (FAO) Codex Alimentarius- Maximum residue limits (mrls) and risk management recommendations (rmrs) for residues of veterinary drugs in foods cac/mrl 2-2015. http://www.agricultura.gov.br/assuntos/inspecao/produtos-animal/plano-de-nacional-de-controle-de-residuos-e-contaminantes/documentos-da-pncrc/codex-alimentarius-cac-mrl-n-o-02-2015-de-julho-2015.pdf/view.

[186] Brazil, Ministério da Agricultura, Pecuária e Abastecimento (MAPA) Instrução normativa no 54, de 17 de dezembro de 2018. http://www.in.gov.br/materia/-/asset_publisher/Kujrw0TZC2Mb/content/id/57733217/do1-2019-01-03-instrucao-normativa-n-54-de-17-de-dezembro-de-2018-57733055.

[187] Brazil, Agência Nacional de Vigilancia Sanitária (ANVISA) Instrução normativa no 51, de 19 de dezembro de 2019. https://alimentusconsultoria.com.br/instrucao-normativa-n-51-de-19-de-dezembro-de-2019-anvisa/.

